# Multiple hetero-redox centers in a phenazine-ketone scaffold for high-energy-density aqueous flow batteries toward durable energy storage

**DOI:** 10.1093/nsr/nwag423

**Published:** 2026-07-20

**Authors:** Zhen Dong, Zhiwen Cui, Hao Fan, Feiyang Hu, Mengke Wen, Yixue Duan, Wenzhang Dong, Chengren Li, Mahalingam Ravivarma, Duanyang Kong, Jiangxuan Song

**Affiliations:** State Key Laboratory for Mechanical Behavior of Materials, Shaanxi International Research Center for Soft Matter, School of Materials Science and Engineering, Xi’an Jiaotong University, Xi’an 710049, China; Shaanxi Coal Chemical Industry Technology Research Institute Co., Ltd., Xi’an 710100, China; State Key Laboratory of Chemical Resource Engineering, Beijing University of Chemical Technology, Beijing 100029, China; State Key Laboratory for Mechanical Behavior of Materials, Shaanxi International Research Center for Soft Matter, School of Materials Science and Engineering, Xi’an Jiaotong University, Xi’an 710049, China; State Key Laboratory for Mechanical Behavior of Materials, Shaanxi International Research Center for Soft Matter, School of Materials Science and Engineering, Xi’an Jiaotong University, Xi’an 710049, China; State Key Laboratory for Mechanical Behavior of Materials, Shaanxi International Research Center for Soft Matter, School of Materials Science and Engineering, Xi’an Jiaotong University, Xi’an 710049, China; State Key Laboratory for Mechanical Behavior of Materials, Shaanxi International Research Center for Soft Matter, School of Materials Science and Engineering, Xi’an Jiaotong University, Xi’an 710049, China; State Key Laboratory for Mechanical Behavior of Materials, Shaanxi International Research Center for Soft Matter, School of Materials Science and Engineering, Xi’an Jiaotong University, Xi’an 710049, China; State Key Laboratory for Mechanical Behavior of Materials, Shaanxi International Research Center for Soft Matter, School of Materials Science and Engineering, Xi’an Jiaotong University, Xi’an 710049, China; State Key Laboratory for Mechanical Behavior of Materials, Shaanxi International Research Center for Soft Matter, School of Materials Science and Engineering, Xi’an Jiaotong University, Xi’an 710049, China; State Key Laboratory of Chemical Resource Engineering, Beijing University of Chemical Technology, Beijing 100029, China; State Key Laboratory for Mechanical Behavior of Materials, Shaanxi International Research Center for Soft Matter, School of Materials Science and Engineering, Xi’an Jiaotong University, Xi’an 710049, China

**Keywords:** redox flow battery, anolyte, hetero-redox center conjugation, multi-electron transfer, high energy density

## Abstract

Organic redox flow batteries operating in alkaline media have attracted significant attention for their inherent safety and environmental compatibility. However, conventional quinone- and ketone-based anolytes suffer from low electron-transfer numbers (≤2 e^−^ per molecule), irreversible isomerization and concentration-dependent side reactions, ultimately limiting their energy density and long-term cycling stability. Here, we introduce a molecular engineering strategy that leverages the conjugation of heterogeneous redox centers to fuse distinct redox-active motifs, thereby creating a new composite phenazine-ketone molecule capable of a six-electron transfer process. This multi-hetero-redox center fused scaffold not only overcomes the intrinsic limitations of conventional structures but also exhibits robust kinetics and chemical stability in an alkaline electrolyte. The resulting compound delivers a record capacity of 79.3 Ah L^−1^, and sustains stable operation over 3500 cycles (198.5 days), offering a viable pathway toward next-generation high-energy-density, durable and sustainable energy storage technologies.

## Teaser

Unlocking reversible six-electron storage in a single conjugated molecule delivers over 3500-cycle stability and high energy density for sustainable aqueous organic flow battery technology.

## INTRODUCTION

Global concerns over fossil fuel depletion and greenhouse gas emissions have intensified the pursuit of sustainable energy sources such as solar and wind power [1,2]. However, the intermittent nature of these renewable resources creates a critical need for large-scale energy storage systems capable of delivering reliable and continuous power [3]. Among the emerging technologies, redox flow batteries (RFBs) have attracted considerable attention due to their inherently scalable architecture, design flexibility and the decoupling of energy and power, enabling cost-effective and durable solutions for grid-scale energy storage [4,5].

Within this context, aqueous RFBs employing organic redox-active materials (ROMs) have emerged as a compelling option, offering intrinsic safety, abundant availability and synthetic tunability [4,6]. Diverse alternatives of ROMs have been developed, drawing on redox-active centers such as quinones [7,8], diimines [9,10], pyridiniums [11,12] and nitroxyl radicals [13,14], with structures optimized for solubility, electrochemical stability and reversibility. Despite this progress, most reported ROMs rely on a single redox-active center and usually support only one-electron transfer per molecule. A few molecules can realize two-electron transfer within one molecule, which offers a route to higher energy density. For example, ketones can undergo sequential two-electron hydrogenation to alcohols [6,15–18], while azines and quinones can complete two proton-coupled electron-transfer processes [19–21]. However, the second electron transfer of ketones often requires more extreme positive potentials [17,18], while the reduced intermediates of azines and quinones are prone to structural rearrangement and degradation, leading to irreversible isomerization and aggregation [22,23]. These factors limit the application of single-center ROMs in high-energy-density and long-lifetime aqueous organic RFBs.

One promising approach is to design ROMs capable of multi-electron transfer (≥3 e^−^ per molecule), thereby increasing charge storage per unit volume [24–26]. Molecular frameworks with multiple identical redox-active centers can, in principle, deliver higher capacities, though practical deployment is often hindered by poor stability. For example, triazine-based ROMs can access four-electron redox states but suffer from poor reversibility due to heteroaromatic core degradation under extreme redox conditions [24]. Asymmetrically structured tetraones with extended π-conjugation have achieved multi-step redox activity with improved radical intermediate stability [25], underscoring the potential of π-delocalized architectures for multi-electron chemistry. Nevertheless, simultaneous realization of the high electron-transfer number, high solubility and long-term stability in aqueous environments has yet to be demonstrated.

Herein, we present a molecular engineering approach that fuses multiple hetero-redox centers into a single π-conjugated framework, yielding composite phenazine-ketone structures capable of six-electron transfer in alkaline aqueous electrolytes and well beyond the capacity of conventional two-electron ketone or phenazine systems. The fused architecture integrates distinct redox moieties to enhance electronic delocalization, stabilize radical intermediates and suppress irreversible isomerization, enabling high ROM concentrations without significantly increasing molecular weight or reducing polarity. As a result, the designed aqueous RFB achieves a high energy density of ~79.3 Ah L⁻^1^ and demonstrates remarkable cycle stability, sustaining over 3500 cycles (198.5 days) under mild conditions. Our findings establish a versatile design blueprint for high-energy-density, durable and environmentally benign ROMs, thereby expanding the molecular toolbox for next-generation aqueous energy storage technologies and addressing critical needs in modern energy infrastructure.

## RESULTS AND DISCUSSION

Fluorenone, renowned for its tunable photophysical and physicochemical properties, serves as versatile building block in diverse applications including antibiotics, anticancer agents, antivirals and neuroregulators [27]. Similarly, natural phenazines, secondary metabolites produced by soil and marine microorganisms such as Streptomyces and Pseudomonas, exhibit strong redox capabilities and are known recognized for their potent antibacterial, antiviral, antimalarial and antitumor activities [28]. Both fluorenone and phenazine frameworks have been increasingly explored as redox-active components for flow battery electrolytes due to their inherent ability to support reversible one- or two-electron transfer processes [17,29,30].

**Figure 1.**
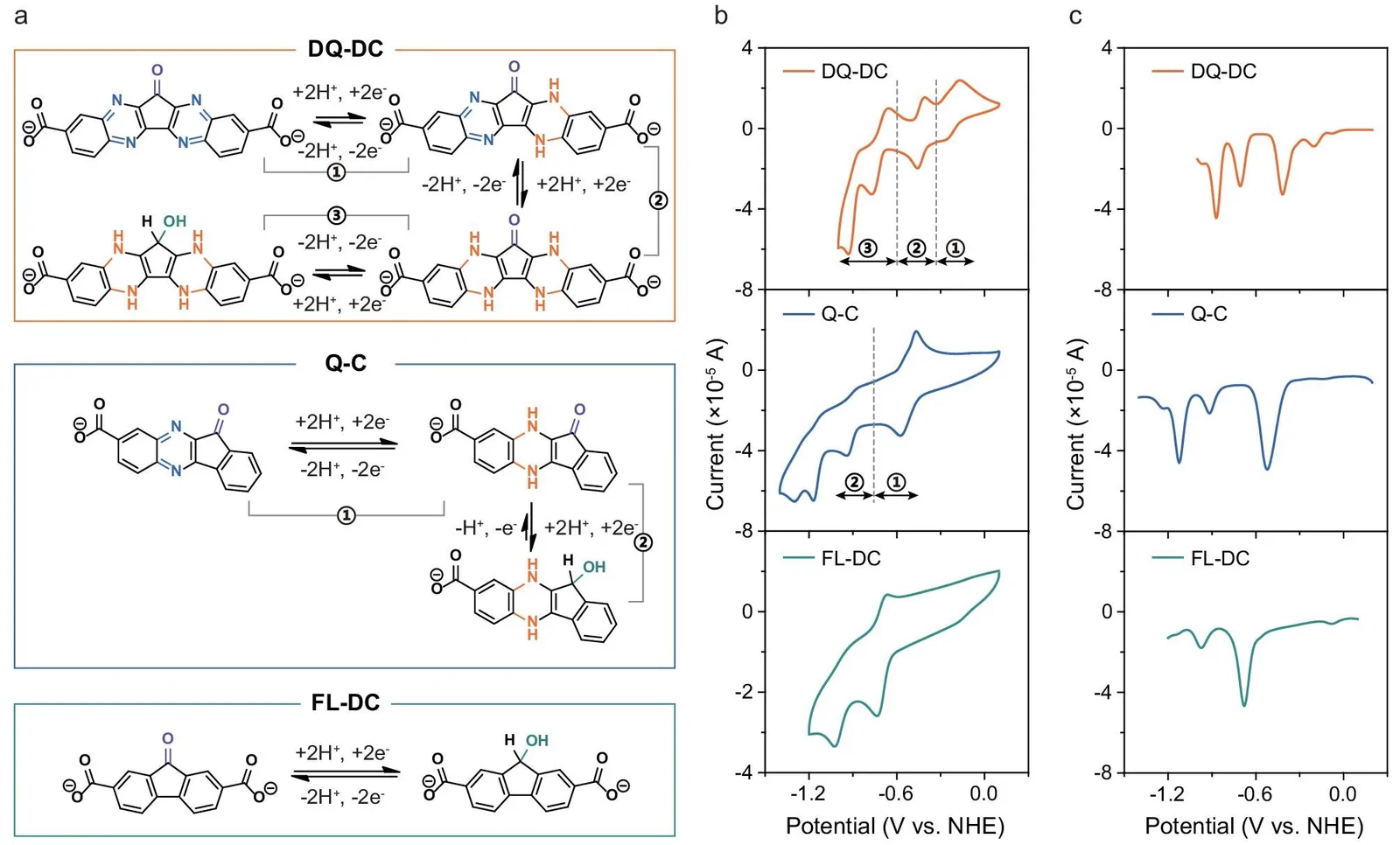
Electrochemical reactions of DQ-DC, Q-C and FL-DC ROMs. (a) Molecular structures with numbered redox centers and the proposed redox mechanisms. (b and c) The corresponding CV (b) and DPV (c) curves. Electrolyte: 4 mM molecules in 1.0 M KOH. Scan rate: 100 mV s^−1^.

Inspired by the complementary redox characteristics of these two molecular classes, we designed and synthesized two fused redox-active structures that integrate the distinct functionalities of fluorene and phenazine moieties. This fusion enables enhanced multi-electron transfer processes mediated by spatially distributed hetero-redox centers. To further enhance compatibility under strongly alkaline conditions, carboxylic acid groups were introduced via molecular engineering, generating two new ROMs designated as DQ-DC and Q-C (Fig. 1a and Figs S1−S5). For comparative analysis, control compound termed FL-DC (Fig. 1a and Figs S6 and S7), containing only the fluorene moiety, was also synthesized to isolate and evaluate the electrochemical behavior intrinsic to the fluorenone unit.

Cyclic voltammogram (CV) measurements conducted in 1.0 M KOH revealed distinct electrochemical signatures for each molecule. DQ-DC displays four well-defined redox peaks centered at ~−0.213, −0.432, −0.718 and −0.899 V versus normal hydrogen electrode (NHE; Fig. 1b and Fig. S8). The Pourbaix diagram demonstrates significant pH dependence of the redox potential, consistent with proton-coupled electron-transfer behavior and indicative of a reversible, four-step, six-electron redox process (Fig. S9). The first two steps correspond to sequential one-electron reductions of the pyrazine moieties, while the subsequent steps are associated with a two-electron redox transformation at the carbonyl group of the fluorenone core [17]. This assignment is supported by semi-*in situ* nuclear magnetic resonance (NMR), electron paramagnetic resonance (EPR) and Raman spectroscopy (Figs S10–S12). Periodic changes in ^1^H nuclear magnetic resonance (NMR) signals, including high-field shifts and the appearance of new resonances, along with systematic variations in EPR radical signals and characteristic functional-group changes in the Raman spectra upon reduction and re-oxidation, collectively corroborate the proposed multi-step mechanism. Differential pulse voltammetry (DPV) further confirmed this behavior, displaying four distinct reduction peaks at −0.204, −0.418, −0.707 and −0.872 V (Fig. 1c).

In contrast, Q-C, featuring a single pyrazine unit fused to a fluorenone structure, is intended to undergo a three-electron transfer process (Fig. 1a). CV and DPV analyses reveal a more mixed electrochemical profile: a pair of reversible redox peaks is observed at ~−0.520 V corresponding to a two-electron transfer process, accompanied by an additional pair of weaker, irreversible peaks at −1.10 V suggesting a less reversible one-electron transition (Fig. 1b and c and Fig. S13). This suggests that the carbonyl group of the fluorenone core may undergo partial or irreversible redox transformations in the absence of stabilizing electron-withdrawing or hydrophilic groups positioned on opposite sides of the ketone core [17,29]. FL-DC, which lacks any pyrazine motif and thus contains only a fluorenone-derived redox center, exhibits a simpler electrochemical response (Fig. 1a). Two pairs of distinct redox peaks are observed at −0.705 and −0.994 V in the CV (Fig. 1b and Fig. S14), which correspond to two DPV reduction peaks at −0.682 and −0.979 V (Fig. 1c). The more straightforward redox signature of FL-DC provides a useful reference point, underscoring the substantial increase in redox complexity and capacity achieved through hetero-redox center integration. Collectively, these results illustrate the power of molecular design in tuning multi-electron transfer characteristics. Comparative electrochemical analyses of DQ-DC, Q-C and FL-DC demonstrate that the strategic incorporation and distribution of redox-active units can significantly enhance electron-transfer capacity while maintaining favorable redox reversibility.

**Figure 2.**
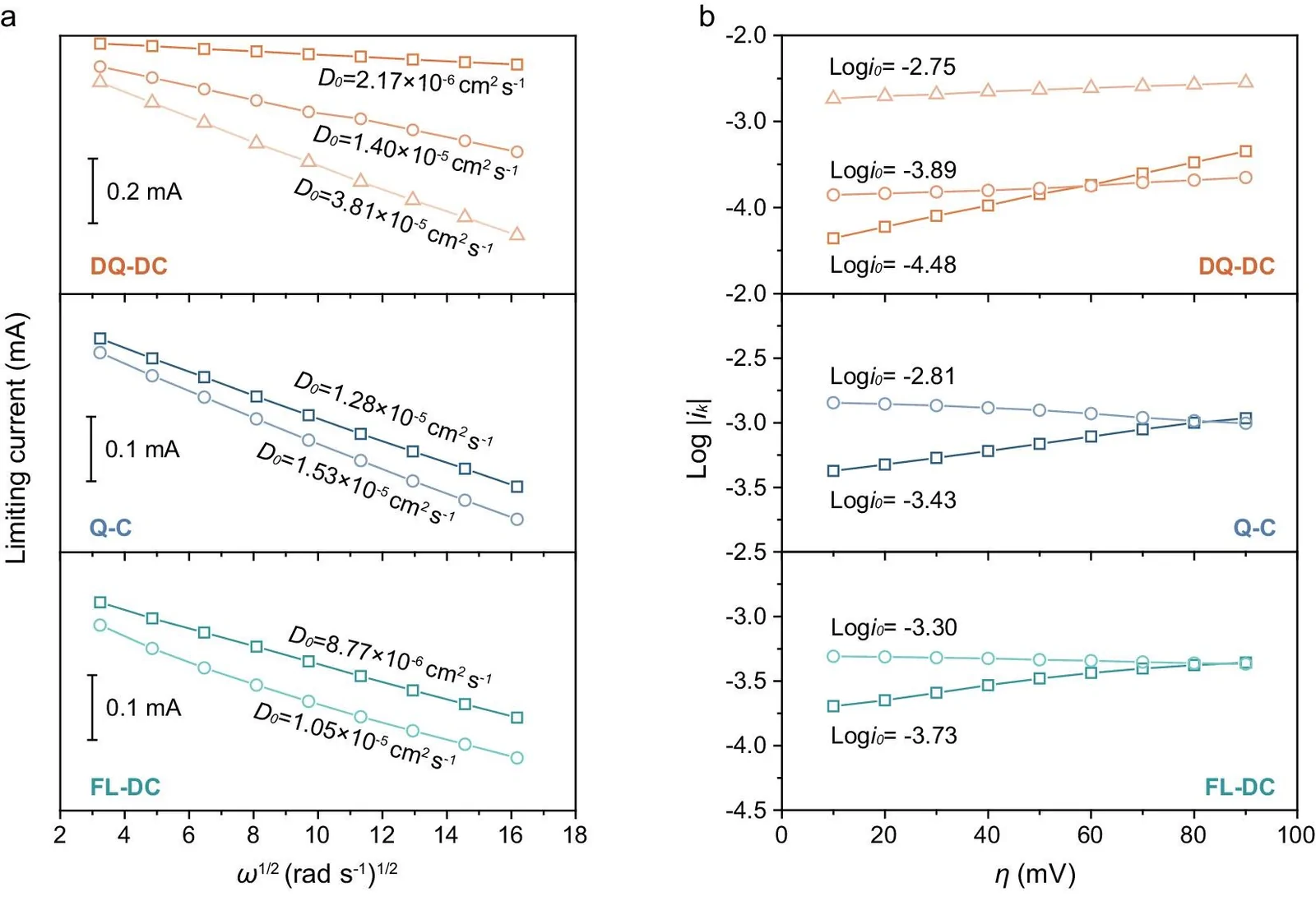
Electrochemical kinetics of DQ-DC, Q-C and FL-DC from LSV studies with 4.0 mM molecules in 1.0 M KOH at a sweep rate of 10 mV s^−1^. (a) Linearly fitted Levich plots of limiting current as a function of the square root of the rotation rates (ω^1/2^). (b) Linearly fitted plots of logarithm of kinetics-controlled current (log*i*_k_) versus overpotential (η).

The electrochemical kinetic of DQ-DC, Q-C and FL-DC were systematically examined using rotating disk electrode (RDE) measurements to evaluate their mass- and charge-transfer characteristics in alkaline electrolyte (Fig. 2). Given the multi-electron redox nature of these molecules, their principal reduction states were treated as discrete redox events for detailed analysis. Linear sweep voltammetry (LSV) curves revealed that the mass transport-controlled limiting currents (*i*_L_) increased with rotation rate, indicating diffusion-dominated transport behavior (Figs S15–S17).

To quantify their mass-transfer kinetics, Levich analyses were performed. The resulting plots of *i*_L_ versus the square root of the rotation rate (ω^1/2^) displayed excellent linearity (Fig. 2a), enabling the determination of diffusion coefficients (*D*₀) for each key redox process. DQ-DC exhibits three main redox steps with *D*₀ of 3.81 × 10⁻^5^, 1.40 × 10⁻^5^ and 2.17 × 10⁻^6^ cm^2^ s⁻^1^, respectively. These values are notably higher than those of Q-C (1.53 × 10⁻^5^ and 1.28 × 10⁻^5^ cm^2^ s⁻^1^) and FL-DC (8.77 × 10⁻^6^ and 1.05 × 10⁻^5^ cm^2^ s⁻^1^), despite DQ-DC’s larger molecular framework. Importantly, the *D*₀ values of DQ-DC substantially exceed those of conventional vanadium redox couples (typically 2.06 × 10⁻^6^ ~3.95 × 10⁻^6^ cm^2^ s⁻^1^ in sulfuric acid and 10⁻^7^~10⁻^6^  cm^2^ s⁻^1^ in theoretical models) [31], and are on par with or surpass several benchmark ROMs such as anthraquinone [32,33], naphthalene diimide [9,34], viologens [35,36] and nitroxide radicals [37,38] underscoring its favorable mass transport properties and efficient molecular delivery to the electrode surface.

Charge-transfer-controlled kinetics, which contribute to the reaction rate of the electrode, were further investigated using the Koutecky–Levich analysis (Figs S15–S17). From the extrapolated kinetic current (*i*_k_) at varying overpotentials (η), Tafel plots of log*i*_k_ versus η were constructed (Fig. 2b), yielding exchange current densities (*i*₀) and kinetic rate constants (*k*₀). DQ-DC exhibits log*i*₀ values of −2.75, −3.89 and −4.48, corresponding to *k*₀ values of 3.66 × 10⁻^2^, 2.65 × 10⁻^3^ and 1.36 × 10⁻^3^ cm s⁻^1^, respectively. In comparison, Q-C displays log*i*₀ values of −2.81 and −3.43 (*k*₀ = 3.18 × 10⁻^2^ and 7.64 × 10⁻^3^ cm s⁻^1^), while FL-DC yields log*i₀* values of −3.30 and −3.73 (*k*₀ = 2.06 × 10⁻^2^ and 7.66 × 10⁻^3^ cm s⁻^1^). Notably, DQ-DC displayed superior kinetic characteristics in terms of both *D*₀ and *k*₀, surpassing a wide range of ROMs including quinones and heterocycles in alkaline media, and viologens and nitroxide radicals in neutral conditions (Table S1). The solubilities of DQ-DC, Q-C and FL-DC in 1.0 M KOH were determined by ultraviolet-visible spectroscopy (UV-Vis) spectroscopy to be 0.57, 0.39 and 0.49 M, respectively (Fig. S18). These outstanding attributes strongly position DQ-DC as a compelling anolyte candidate for high-performance aqueous RFBs (Table S2).

**Figure 3.**
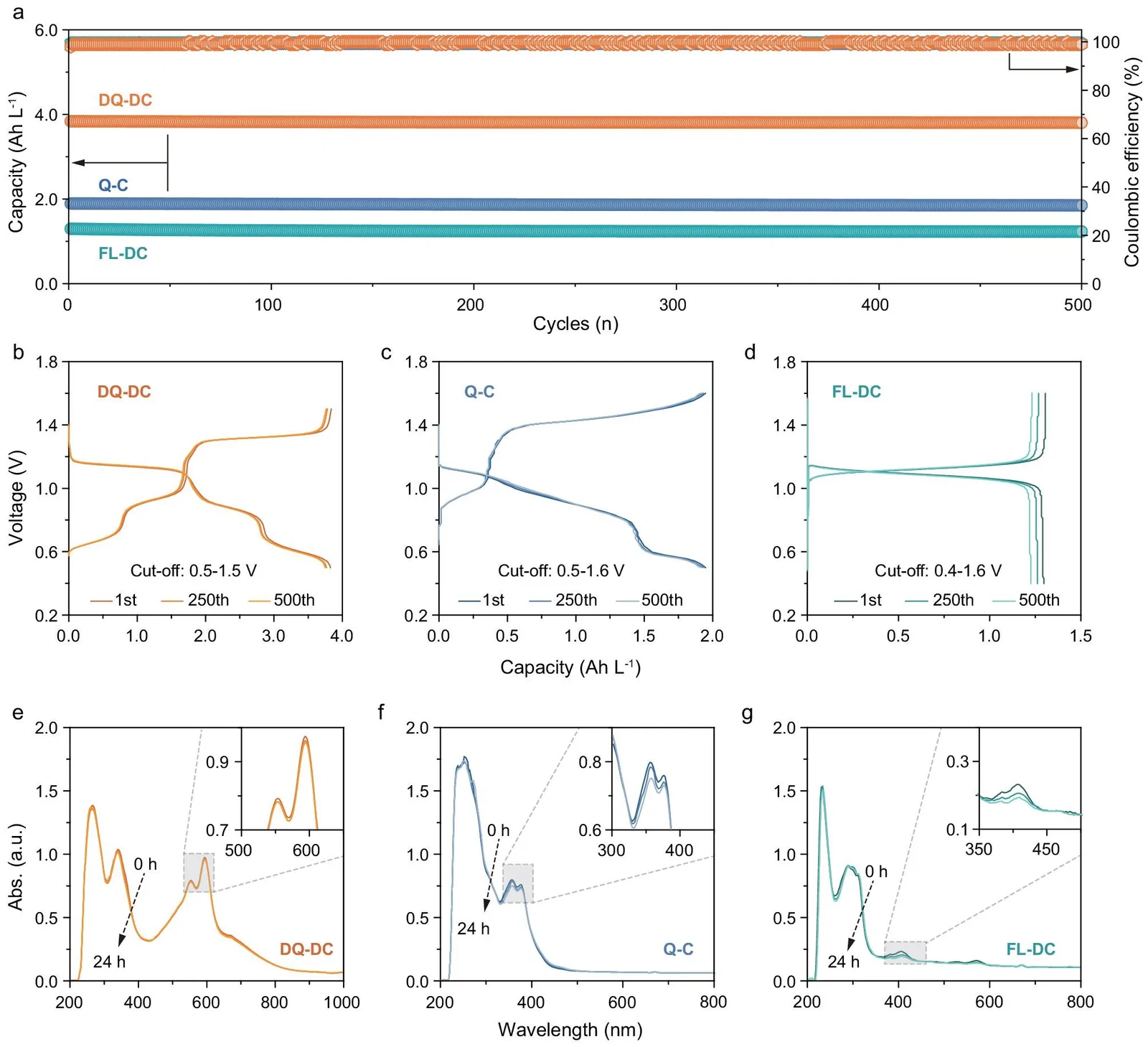
Electrochemical performances of DQ-DC-, Q-C- and FL-DC-based RFBs. (a) Cycling capacities with respect to cycle numbers. (b–d) Representative charge–discharge voltage profiles of the three molecule-based RFBs. (e–g) The corresponding time-dependent *in situ* UV-Vis spectra of the reduction form at 100% SOC. Insets are the enlarged views of absorption variation at typical peaks of the three molecules.

The battery performance of DQ-DC, Q-C and FL-DC anolytes was systematically evaluated using a custom-built RFB platform (Fig. 3). Long-term cycling tests were conducted to assess the stability and capacity retention rate of these ROMs. Each anolyte was used at 0.025 M concentration in 1.0 M KOH, corresponding to total electron-transfer concentrations of 0.15 M (DQ-DC, 6e⁻), 0.075 M (Q-C, 3e⁻) and 0.05 M (FL-DC, 2e⁻). A designed K_4_Fe(CN)_6_ solution in 1.0 M KOH was employed as the catholyte to maintain K^+^ ion balance and osmotic equilibrium across the Nafion membrane. All systems exhibit consistently high Coulombic efficiencies (CEs) of ~99.5% over extended cycling, indicating excellent charge/discharge reversibility and minimal parasitic side reactions (Fig. 3a). Among the three, DQ-DC delivers the highest volumetric discharge capacity (DC) of ~3.8 Ah L⁻^1^, corresponding to ~95% of its theoretical six-electron capacity with an exceptionally low fade rate of 0.0018% per cycle and 0.038% per day. By comparison, Q-C and FL-DC achieve capacities of ~1.9 and ~1.3 Ah L⁻^1^, corresponding to ~95% and ~96% of their theoretical capacities with the fade rates of 0.0046% per cycle and 0.21% per day for the former and 0.011% per cycle and 0.78% per day for the latter.

Representative charge–discharge voltage profiles were recorded during the 1st, 250th and 500th cycles at 20 mA cm⁻^2^ (Fig. 3b–d). The DQ-DC-based RFB displays three distinct and stable charge/discharge voltage plateaus at 0.62/0.57 V, 0.93/0.88 V and 1.33/1.15 V (Fig. 3b), which align well with the redox potentials identified in CV and DPV analyses (Fig. 1b). The comparable lengths of these plateaus suggest that the four sequential redox steps of the six-electron transfer process contribute nearly equally to the total capacity. In contrast, the Q-C-based RFB exhibits two voltage plateaus at 0.97/0.57 V and 1.43/0.96 V (Fig. 3c), the second plateau being significantly shorter (approximately one-third of the first), suggesting asymmetric contribution from the redox steps [30]. Only a single charge/discharge plateau is observed, indicating partial redox irreversibility or incomplete recovery of the charged state. The FL-DC-based RFB demonstrates two well-defined voltage plateaus at 0.68/0.62 V and 0.93/0.87 V (Fig. 3d), consistent with its expected two-electron redox behavior, and showed good electrochemical stability across cycles.

To further probe redox-state stability, *in situ* UV-Vis spectra were performed at 100% state of charge (SOC). DQ-DC, Q-C and FL-DC reveal distinct absorption features centered at 550–600 nm, 350–400 nm and 350–450 nm, respectively (Fig. 3e–g). These spectral signatures remain largely unchanged over a 24-hour open-circuit rest period, indicating strong redox-state persistence. The relative changes in peak intensity follow the trend FL-DC (1.9%) > Q-C (2.3%) > DQ-DC (5.5%), suggesting superior long-term redox stability of DQ-DC under idle conditions. Taken together, these results demonstrate that DQ-DC not only exhibits outstanding cycling stability and multi-electron utilization, but also delivers ~2–3 times as much as the DC of Q-C and FL-DC under identical conditions.

**Figure 4.**
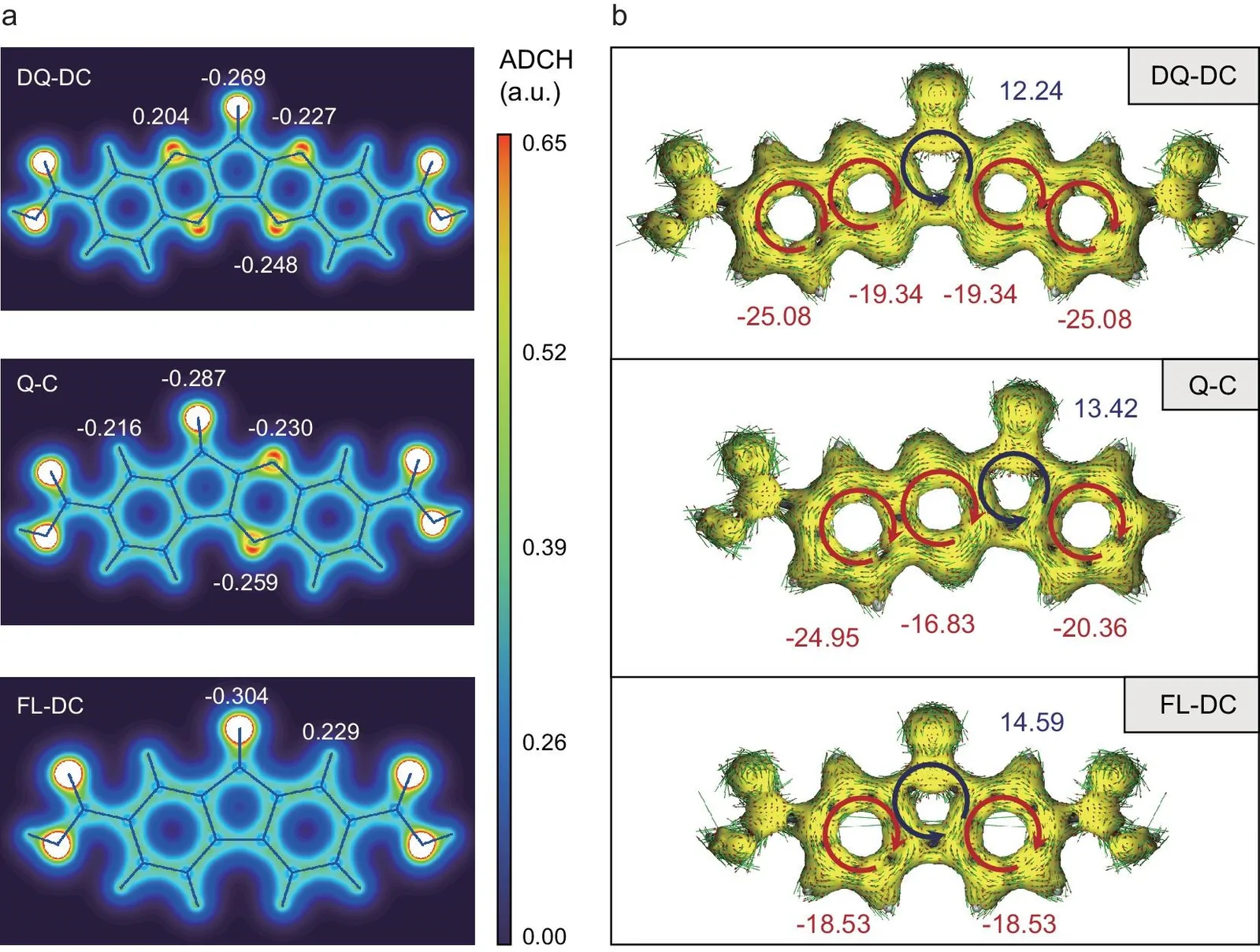
Theoretical analyses of DQ-DC, Q-C and FL-DC. (a) Calculated ADCH atomic charge values. (b) Calculated AICD plots and corresponding NICS(1)_ZZ values. The plane with circles marked for DQ-DC, Q-C and FL-DC was placed perpendicular to the magnetic field vector. Small green arrows show the computed current density vectors. The circles marked with red and blue arrows indicate the clockwise (aromaticity) and anticlockwise (antiaromaticity) ring current flows, respectively. NICS(1)_ZZ refers to the negative magnetic shielding value measured perpendicular to the ring plane at a distance of 0.1 nm above it; thus, a negative NICS value indicates aromatic character [40,44].

A quantitative understanding of the relationship between molecular structure and electrochemical performance requires elucidation of how heteroaromatic ring fusion modulates electron density and delocalization. To this end, we employed atomic dipole moment-corrected Hirshfeld (ADCH) charge analysis, electrostatic potential (ESP) mapping and combined anisotropy of the induced current density (AICD) and nucleus-independent chemical shift (NICS) calculations to reveal structure–property correlations at the electronic level [39,40]. This integrated approach preserves molecular dipole moments while partitioning electron density into atomic contributions, enabling chemically intuitive visualization of charge distribution and aromaticity. The resulting atomic charges show strong correlation with both ESP surfaces and experimental dipole moments. For the unfused FL-DC molecule, ADCH analysis reveals a pronounced polarization of the carbonyl group, with partial charges of +0.229 (C) and −0.304 (O) on the C=O bond (Fig. 4 and Fig. S19). Incorporation of a single pyrazine ring in Q-C reduces these values to +0.216 and −0.287, respectively, while DQ-DC, featuring two fused pyrazine rings, further attenuates them to +0.204 and −0.269. This progressive decrease highlights enhanced π-delocalization over the extended conjugated backbone [39,41]. Simultaneously, the N atoms within the pyrazine moieties shift from −0.259/−0.230 in Q-C to −0.248/−0.227 in DQ-DC, indicating redistribution of negative charge across the fused framework [42]. ESP mapping provides a three-dimensional visualization of these effects (Fig. S19). For FL-DC, deep negative potential wells are localized near the carbonyl oxygen atoms, consistent with their high partial charges. In contrast, Q-C and DQ-DC exhibit more diffuse and uniformly distributed ESP features, particularly across the pyrazine bridges, reflecting increased electronic delocalization and a smoother electrostatic landscape [43].

To further assess aromaticity and current delocalization, we analyzed AICD contour plots in combination with NICS(1)_ZZ values (Fig. 4b). In DQ-DC, both the benzene and pyrazine rings support strong diatropic (clockwise) ring currents, indicative of pronounced aromatic character. The benzene rings possess particularly high aromaticity, with NICS(1)_ZZ values of −25.08, while the pyrazine rings display significant aromaticity at −19.34. In Q-C, the two benzene rings yield NICS(1)_ZZ values of −24.95 and −20.36, while the single pyrazine ring shows a slightly diminished aromaticity (−16.80). In FL-DC, the two isolated benzene rings exhibit lower aromaticity (−18.53), consistent with the lack of extended conjugation.

Interestingly, analysis of the central fluorenone subunit reveals a progressive decrease in antiaromatic character across the three molecules, with NICS(1)_ZZ values of 14.59 for FL-DC, 13.42 for Q-C and 12.24 for DQ-DC. The combination of enhanced aromatic stabilization in the fused rings and diminished antiaromaticity in the fluorenone core suggests that DQ-DC achieves a particularly favorable balance of electronic delocalization, contributing to its superior thermodynamic stability. Collectively, these findings reveal that heterocycle fusion modulates both local bond polarization and global electrostatic environments in ROMs.

**Figure 5.**
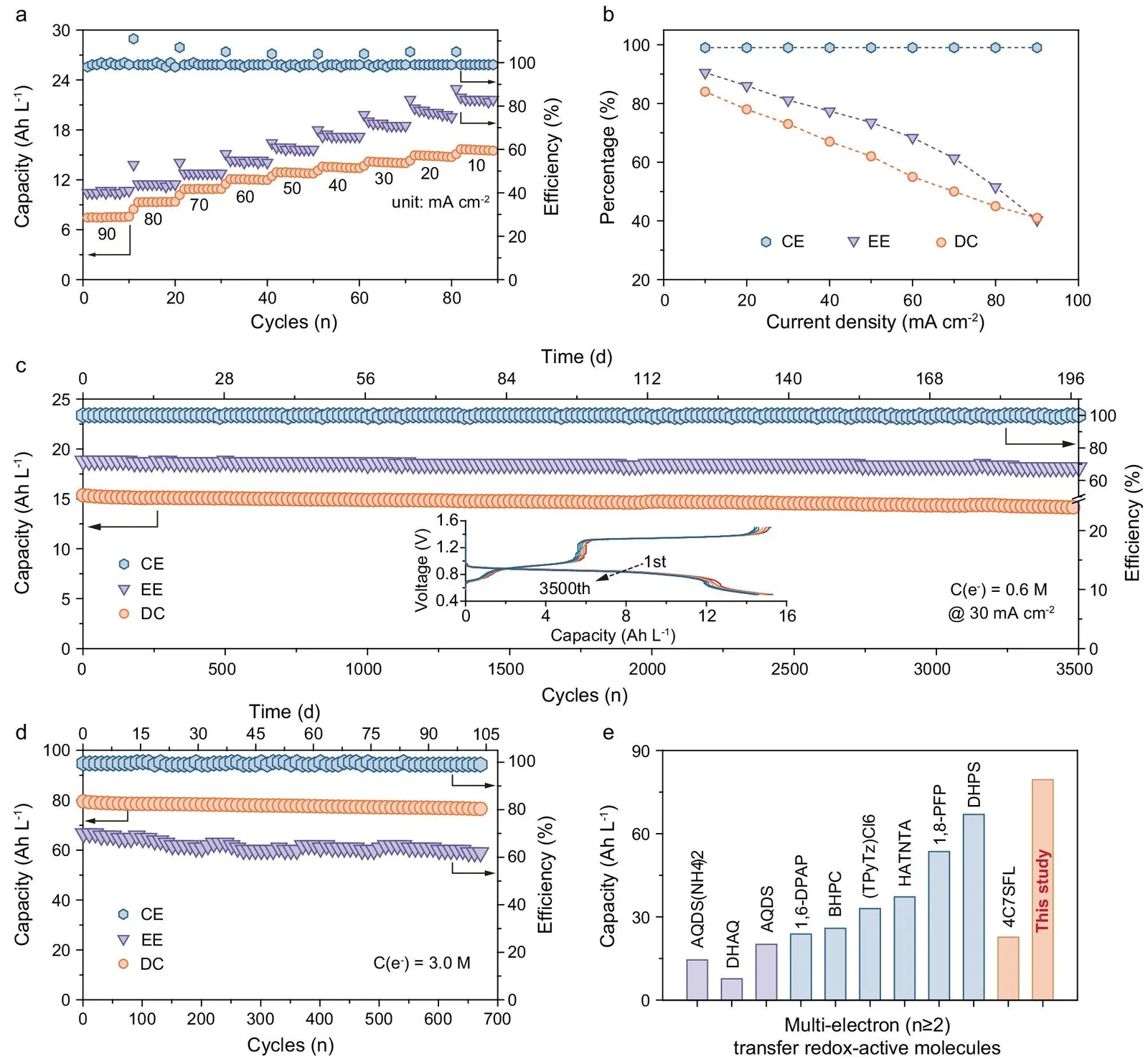
Battery performance of the DQ-DC-based RFBs. (a) Capacity versus the cycles from 10 to 90 mA cm^-2^ of the battery. Ten cycles’ test was conducted for each current density. (b) CE, CU and round-trip EE versus current density. (c) DC, CE and EE versus time and cycle number. The battery operation across 198.5 days. Inset is the polarization curve at selected cycles. (d) High-concentration battery demonstrated with 0.5 M DQ-DC. (e) The capacity comparison of quinones-, azine- and fluorenones-based molecules with multi-electron utilized in RFBs in terms of ampere-hours per liter [17,22,24,26,33,45–49].

The rate capability of the 0.1 M DQ-DC-based aqueous RFB was systematically evaluated over a wide current density range (Fig. 5a and b and Fig. S20). As the current density was stepped from 10 to 90 mA cm⁻^2^, the DC decreased steadily from 15.6 to 7.5 Ah L⁻^1^ (Fig. 5a), corresponding to utilization rates of 97.1%, 92.6%, 87.7%, 83.9%, 80.1%, 74.8%, 67.8%, 58.1% and 46.7%, respectively (Fig. 5b). Despite this progressive decline in capacity—primarily attributable to increased mass transport and kinetic limitations—the CE remained close to 100% across all current densities, while energy efficiency (EE) decreased from ~84% to ~41% due to increasing ohmic polarization, as evident in the voltage profiles (Fig. S20). At 100 % SOC, the peak power density of the battery reaches 250.2 mW cm^−2^ at a current density of 100 mA cm^−2^ (Fig. S21). To probe long-term stability, galvanostatic cycling was performed at 30 mA cm⁻^2^ between 0.5 and 1.5 V for 3500 cycles (198.5 days; Fig. 5c). Impressively, the battery preserves a remarkable ~92% of its initial capacity with a minimal fade rate of 0.0023% per cycle (0.039% per day), while maintaining nearly 100% CE and a stable EE of ~68%.

The charge–discharge curves (inset, Fig. 5c) show three distinct plateaus at 0.75/0.55 V, 0.93/0.78 V and 1.35/0.87 V, corresponding to two consecutive two-electron redox events followed by a third two-step two-electron process, in full agreement with the multi-step redox mechanism derived from CV and LSV analyses (Fig. 1b and Figs S8, S9 and S15). Encouraged by the superior cycling stability and good solubility, we further increased the DQ-DC anolyte concentration to 0.5 M to enhance energy density. Operated at 50 mA cm⁻^2^, the concentrated RFB sustained 700 cycles over 108.6 days, delivering a volumetric capacity of 79.3 Ah L⁻^1^ with a modest decay of 0.0063% per cycle and 0.042% per day (Fig. 5d and Fig. S22). Post-cycling NMR analyses reveal no detectable decomposition products (Fig. S23), and time-dependent quantitative analysis indicates an average molecular degradation rate of ~0.033% per day (Fig. S24). The post-cycle CV profile of anolyte retains well-defined oxidation and reduction peaks that are characteristic of the DQ-DC (Fig. S25). In contrast, the CV of the catholyte displays minor features that may be attributable to a small degree molecular crossover from the anolyte compartment (Fig. S26). The DQ-DC anolyte was then dissolved in methanol and analyzed by electrospray ionization mass spectrometry (ESI-MS) in negative-ion mode (Fig. S27). For the pristine sample, only the intact molecular ions [M + CH₃OH − H]⁻ and [M + CH₃OH]⁻ were observed at m/z = 403.0684 and 404.0717, respectively, with no additional fragments detected. In contrast, the cycled anolyte exhibits prominent peaks at m/z = 1155.4285 and 1156.4318, which we tentatively assign to a tetrameric species formed via decarboxylation of the reduced state. Such minor permeation and the formation of aggregated species are plausible given the extended duration of operation and may contribute to the gradual capacity decay observed over long-term cycling. These results represent the highest practical-to-theoretical capacity utilization ratios reported to date for multi-electron organic anolytes in aqueous RFBs (Fig. 5e and Table S3).

One of the unique advantages of the DQ-DC-based RFB lies in its capacity for real-time, visual SOC estimation via distinct and progressive color changes of the redox-active species. Accurate SOC monitoring is critical for RFBs to prevent operational hazards such as gas evolution [50], and becomes even more essential in large-scale applications, such as grid-scale energy storage system, where it informs users when to recharge and helps prevent overcharging [30]. Remarkably, DQ-DC undergoes vivid and reversible color transitions, from yellow to green, purple and ultimately red, across different SOC levels, enabling simple and rapid visual feedback.

**Figure 6.**
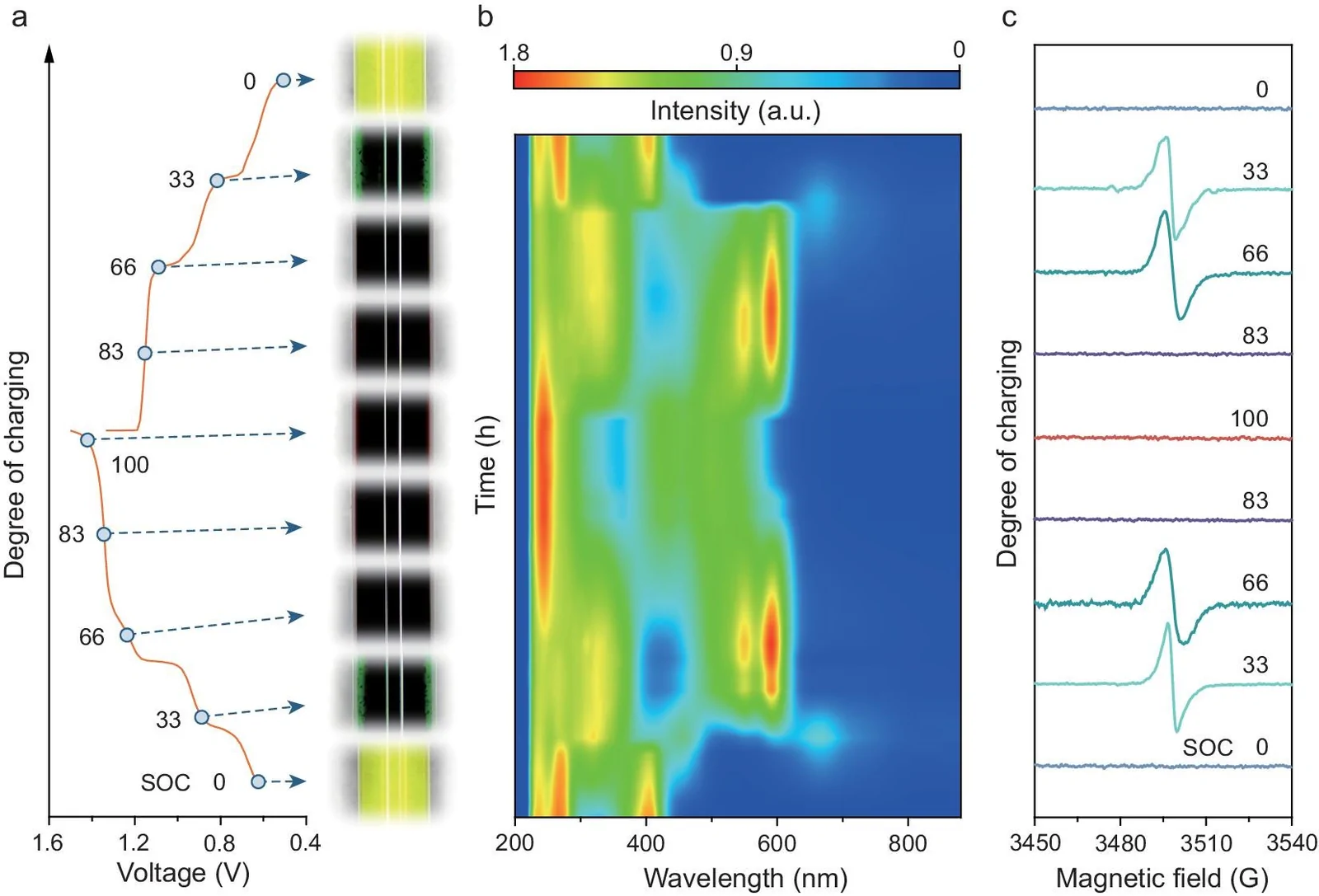
Prediction of SOC for DQ-DC. (a and b) Analysis of color changes as a function of the SOC using *in situ* UV-Vis spectroscopy; the dotted arrows point to the corresponding colors of the electrolytes in the anolyte compartment. (c) The corresponding EPR spectra.

To characterize this chromatic response, *in situ* UV-Vis spectra and corresponding color were recorded in the anolyte compartment as a function of SOC, using ~16% increments (Fig. 6a). Four distinct color stages emerge during the charging process, aligning with the stepwise multi-electron redox reactions of DQ-DC. In the initial stage (SOC 0%–33%), the yellow solution progressively turns green as DQ-DC was reduced to DQ-DC-2H, accompanied by decreasing absorbance at 244 and 404 nm (Fig. 6b and Figs S28 and S29). In the second stage (SOC 33%–66%), the green hue transforms into purple, with increasing intensity of absorption bands at 318, 550 and 590 nm, reflecting the formation of DQ-DC-4H. In the third stage (SOC 66%–83%), the purple gradually shifts to dark red as absorbances at 318, 550 and 590 nm declined, while signals near 244 nm increased. Finally, in the last stage (SOC 83%–100%), minimal color and spectral changes are observed. These spectral transitions were fully reversible during discharge, reinforcing their correlation with redox-state evolution.

Although EPR spectroscopy has previously been explored for SOC determination in RFBs [51], it is impractical for routine implementation—especially in multi-electron ROM systems where radical concentrations do not correlate linearly with SOC (Fig. 6c). In contrast, the multi-step redox mechanism of DQ-DC enables robust optical tracking. Four representative absorption peaks at 318, 404, 550 and 590 nm were selected as markers for quantitative UV-Vis-based SOC determination. Strong linear correlations between peak absorbance and SOC are observed across multiple redox stages (Fig. S30), demonstrating that precise and real-time SOC analysis can be reliably achieved in the DQ-DC system through simple UV-Vis spectroscopic monitoring.

## CONCLUSION

The rational design of multi-electron ROM anolytes that combine high redox capacity, long-term cycling stability and good aqueous compatibility remains a key challenge in advancing aqueous organic RFBs. Here, we present a molecular engineering strategy that integrates two distinct redox-active motifs, quinone and pyrazine, into a single π-conjugated framework to yield a phenazine-ketone-based molecule capable of undergoing a six-electron redox process. The resulting compound demonstrates high aqueous solubility, robust redox kinetics and remarkable chemical stability under alkaline conditions. When integrated in an RFB configuration with K_4_[Fe(CN)_6_] as the catholyte, the resulting system delivers a record-high capacity of ~79.3 Ah L⁻^1^ and demonstrates exceptional long-term durability over prolonged cycling over 3500 cycles (198.5 days) with negligible capacity decay. This study showcases the effectiveness of conjugating multiple hetero-redox centers to simultaneously address solubility, stability and capacity trade-offs. It further provides a compelling molecular blueprint for the development of safe, cost-effective and scalable ROMs tailored for high-energy-density and long-duration energy storage applications.

## METHODS

### Synthesis processes

DQ-DC was synthesized according to reported procedures [52,53] from leuconic acid and 3,4-diaminobenzoic acid in refluxing AcOH, followed by oxidation with 30% HNO_3_ at 140°C, giving a yellow solid in 60% yield. Q-C was prepared following a literature method [54] by condensing 2,2-dihydroxyindane-1,3-dione with 3,4-diaminobenzoicacid in refluxing EtOH, affording 85% yield. FL-DC was obtained according to previous reports [55,56] through Friedel–Crafts acetylation of fluorene with Ac_2_O/AlCl_3_, then oxidation with NaOCl and acidic hydrolysis, delivering yields of 61% and 85% over two steps. All products were characterized by ^1^H and ^13^C NMR.

### Electrochemical measurements

CV and LSV with RDE were performed on an electrochemical workstation using a three electrodes system. A glassy carbon working electrode was polished and rinsed before each measurement. A Pt sheet and saturated Ag/AgCl served as the counter and reference electrodes, respectively. CV curves were recorded at scan rates of 9–625 mV s⁻^1^ with 2.0 mM active materials in 1.0 M KOH, while LSV polarization curves were obtained at rotation speeds of 100–2500 rpm at 10 mV s⁻^1^ after deoxygenation with N_2_. All potentials were calibrated with potassium ferricyanide and converted to the NHE.

### Battery tests

Battery tests were conducted under a Neware battery test system (CT-4008-5V6A-S1-F, Neware, Shenzhen, China). A lab-scale flow cell was assembled with graphite end plates, Cu current collectors, graphite felts and a cation exchange membrane, with an active area of 5.0 cm^2^. The electrolyte materials at the same concentration were dissolved in 1.0 M KOH. The anolytes contain DQ-DC, Q-C or FL-DC, while the catholyte is composed of K_4_Fe(CN)_6_ in 1.0 M KOH. All batteries were evaluated for multi-electron storage as detailed earlier. Electrolytes were pumped into the cell at 60 mL min⁻^1^ using a peristaltic pump. All battery measurements were conducted in an Ar-filled glovebox at room temperature (~25 °C) to ensure an inert and controlled testing environment.

### Density functional theory calculations

All density functional theory calculations were performed with the Gaussian 16. Geometry optimizations and frequency calculations were carried out with the B3LYP functional with 6-311+G(d, p) basis set [57,58]. The D3 dispersion correction was added to ensure proper description of dispersive interactions. The implicit solvation model based on density (SMD) solvent model was used to represent the solvent effect of water molecule [59,60]. ADCH charge and electron-density analyses were undertaken with the Multiwfn program [39,61,62]. NICS were calculated at the same level according to the procedure described by Jonathan R. Nitschke [63]. NICS(1)zz is the negative value of the shielding tensor component perpendicular to the ring plane at 1 Å above the ring plane. ACID plots were generated at the B3LYP/6-31G(d) level according to the method of Herges [40], with visualization carried out by using POV-Ray v3.7 software.

## Supplementary Material

nwag423_Supplemental_File
