# Supplementary material for: Multiple hetero-redox centers in a phenazine-ketone scaffold for high-energy-density aqueous flow batteries toward durable energy storage
Source: Natl Sci Rev. 2026 Jul 20;13(16):nwag423. doi: 10.1093/nsr/nwag423 (PMC13525713; doi:10.1093/nsr/nwag423)
Supplement: Supplementary file 1 [file nwag423_supplemental_file.pdf]

Supplementary information

Multiple Hetero-Redox Centers in a Phenazine-Ketone Scaffold for High-Energy-Density Aqueous Flow Batteries Toward Durable Energy Storage

Zhen Dong[1,2,†], Zhiwen Cui[3,†], Hao Fan[1,*], Feiyang Hu[1], Mengke Wen[1], Yixue Duan[1], Wenzhang Dong[1], Chengren Li[1], Mahalingam Ravivarma[1], Duanyang Kong[3,*] and Jiangxuan Song[1,*]

[1]State Key Laboratory for Mechanical Behavior of Materials, Shaanxi International Research Center for Soft Matter, School of Materials Science and Engineering, Xi'an Jiaotong University, Xi'an 710049, China;

[2]Shaanxi Coal Chemical Industry Technology Research Institute Co., Ltd., Xi'an 710100, China;

[3]State Key Laboratory of Chemical Resource Engineering, Beijing University of Chemical Technology, Beijing 100029, China

***Corresponding authors.** E-mails: fanh2018@xjtu.edu.cn; kongdy@buct.edu.cn; songjx@xjtu.edu.cn

†Equally contributed to this work.

Table of Contents

Fig. S1. Synthetic route illustrations for DQ-DC. 3

Fig. S2. ^1H NMR spectrum of DQ-DC, recorded in DMSO-d_6. 4

Fig. S3. Synthetic route of Q-C. 5

Fig. S4. ^1H NMR spectrum of Q-C, recorded in DMSO-d_6. 5

Fig. S5. Synthetic route of FL-DC. 5

Fig. S6. ^1H NMR spectrum of FL-DC, recorded in D_2O. 6

Fig. S7. CV analyses for DQ-DC in 1.0 M KOH. 7

Fig. S8. CV analyses for Q-C in 1.0 M KOH. 8

Fig. S9. CV analyses for FL-DC in 1.0 M KOH. 9

Fig. S10. LSV analyses for DQ-DC in 1.0 M KOH. 10

Fig. S11. LSV analyses for Q-C in 1.0 M KOH. 11

Fig. 12. LSV analyses for FL-DC in 1.0 M KOH. 12

Table S1. The comparison of mass- and charge-transfer-controlled kinetic parameters of DQ-DC and previously reported redox-active materials in RFBs. 13

Fig. S13. The ESP interpenetration diagrams of DQ-DC, Q-C, and FL-DC. 15

Fig.S 14 Charge-discharge voltage profiles of 0.1 M DQ-DC-based RFB. 16

Fig. S15. Battery voltage versus charge-discharge curves of the 0.5 M DQ-DC-based RFB. 17

Fig. S16. Post-cycling permeability analysis of $K_4Fe(CN)_6$ catholyte. 18

Fig. S17. Post-cycling characterization of the 0.5 M DQ-DC anolyte. 19

Fig. S18. In-situ UV-Vis spectra of the DQ-DC anolyte recorded during stepwise charging from 0% to 100% SOC. 20

Fig. S19. The photographs of DQ-DC in 1.0 M KOH at varying SOCs. 21

Fig. S20. Linearly fitted plot of the absorbance at 318, 404, 550 and 590 nm as a function of the SOC. 22

Table S2. The comparison of electrolytes and battery performances of the phenazine-based RFBs. 23

References 25

Chemicals and materials

All chemicals were used as received without any further purification. Deionized water (DI H_2O) was used throughout the study. Leuconic acid, 3,4-diaminobenzoic acid, chloroacetyl chloride, 2,2-dihydroxyindane-1,3-dione, fluorene, tetrabutylammonium bromide were purchased from Energy Chemical Co., Ltd. (Shanghai, China). Acetic acid (AcOH), ethyl alcohol, acetic anhydride, $AlCl_3$, 1,1,2,2-tetrachloroethylene, sodium hydroxide, dimethyl sulfoxide, sodium hypochlorite solution was obtained from Sinopharm Chemical Reagent Co., Ltd. (Shanghai, China). All reactions were monitored by TLC analysis. Cation-exchange membrane (CEM, counter ion: K^+, thickness: 125 μm) was produced from Suzhou Thinkre New Materials Co., Ltd. (Suzhou, China). Cu current collectors and graphite felt were taken from JING LONG TE TAN Tech Co., Ltd. (Beijing, China). BT300M peristaltic pumps were acquired from Innofluid Co., Ltd. (Shanghai, China).

Synthesis processes

DQ-DC was synthesized according reported procedures with minor modifications.(1,2) Under nitrogen atmosphere, a mixture of leuconic acid **1** (0.420 g, 3.0 mmol) and 3,4-diaminobenzoic acid **2** (1.004 g, 6.6 mmol) was dissolved in AcOH (45 mL) and reflux for 8 h. The resulting dark suspension was filtered and the solid was washed sequentially with water, ethanol, and acetone. The obtained solid was then suspended in 30 wt.% HNO_3 (97 mL) and stirred at 140 °C for 3 h. After cooling, the suspension was filtered, and the collected solid was washed with water and ethanol, and subsequently dried under vacuum to afford a yellow solid (0.670 g, yield 60%). ^1H NMR (600 MHz, DMSO-d_6) δ: 8.79 – 8.77 (m, 2H), 8.49 – 8.44 (m, 2H), 8.44 – 8.42 (m, 2H). ^{13}C NMR (151 MHz, DMSO-d_6) δ: 130.7, 130.8, 131.3, 131.4, 131.5, 131.5, 132.4, 132.4, 133.8, 133.9, 134.7, 141.8, 141.9, 142.4, 142.5, 144.3, 144.4, 144.8, 144.9, 151.6, 151.7, 152.1, 152.2, 154.2, 154.3, 154.8, 154.9, 166.1, 166.2, 185.9, 185.9.

Q-C was synthesized according reported procedures with minor modifications.(3) Under nitrogen atmosphere, a mixture of 2,2-dihydroxyindane-1,3-dione **3** (1.522 g, 10.0 mmol) and 3,4-diaminobenzoicacid **2** (1.960 g, 11.0 mmol) was dissolved in EtOH (250 mL) and heated to reflux for 10 h. The pale green suspension was filtered and washed with ethanol, then dried under vacuum to afford a pale green solid (2.348 g, yield 85%). ^1H NMR (400 MHz, DMSO-d_6) δ 8.64 (d, J = 1.7 Hz, 1H), 8.34 (dd, J = 8.6, 1.9 Hz, 1H), 8.22 (d, J = 8.7 Hz, 1H), 8.14 (d, J = 7.5 Hz, 1H), 7.96 – 7.89 (m, 2H),

7.79 – 7.74 (m, 1H).

FL-DC was synthesized according to previous reports.(4,5) $AlCl_3$ (43.7 g, 327 mmol) was dissolved in 1,1,2,2-tetrachloroethylene (48 mL) and stirred at 0 °C. Acetic anhydride (Ac_2O, 15.5 mL, 16.8 g, 165 mmol) was added dropwise at 0 °C. The mixture was then allowed to warm to room temperature (25 °C) and subsequently added dropwise to a stirred solution of fluorene **4** (8.00 g, 48.1 mmol) in 1,1,2,2-tetrachloroethylene (48 mL). The reaction mixture was heated to reflux in an oil bath and stirred for 1.5 h. After cooling to room temperature, the mixture was poured into crushed ice. Once the ice had melted, CH_2Cl_2 (775 mL) was added, and the aqueous phase was acidified to pH=1 with 2 N HCl. The organic layer was separated, and the aqueous layer was extracted with CH_2Cl_2 (2×75 mL). The combined organic extracts were washed with brine, dried over anhydrous $MgSO_4$, filtered, and concentrated under reduced pressure. The crude product was recrystallized from acetone and dried under vacuum to afford 2,7-diacetylfluorene as pale yellow crystals **5** (7.33 g, yield 61%). 2,7-diacetylfluorene **5** (5.0 g, 0.02 mol), sodium hydroxide (8.0 g, 0.20 mol) and tetrabutylammonium bromide (TBAB, 0.5 g) was dissolved in dimethyl sulfoxide (DMSO, 200 ml), sodium hypochlorite solution (50.0 g, activated chlorine content = 10%) was added slowly at 10 °C. The mixture was stirred for 2 h at room temperature and then heated to 70 °C for 4 h. The sodium hyposulfite was added to quench the excess sodium hypochlorite, then the concentrated hydrochloric acid (37%, 30 ml) was added. The crude product was recrystallized from ethanol and dried under vacuum to afford 2,7-dicarboxyl fluorenone as yellow crystals FL-DC (4.56 g, yield 85%). ^{1}H NMR (600 MHz, D_2O) δ 7.65 (d, J = 7.5 Hz, 2H), 7.41 (s, 2H), 6.92 (d, J = 7.5 Hz, 2H).

Characterizations

Ultraviolet-visible (UV-Vis) spectra were measured using a spectrometer UV-3600 plus (Shimadzu Scientific Instrument) with a slit width of 1.0 nm and quartz spectrophotometer cell (Aldrich, 10 mm of optical path length). The spectrum was measured at a wavelength range of 200-700 nm at a sampling interval of 0.2 nm. Electrospray ionization mass spectra (ESI-MS) were collected by using WATERS I-Class VION IMS QTof Mass Spectrometer in negative ion mode. Nuclear magnetic resonance (NMR) spectra were measured on a Bruker Avance-III HD 400 or 600 MHz spectrometer. Electron paramagnetic resonance (EPR) spectra were conducted at 298

K on a Bruker A300 EPR spectrometer. The pH value of each electrolyte was measured with an Oakton pH meter (pH-2700). Raman spectra were recorded on a Thermo Scientific DXR3xi Raman imaging microscope. The excitation light of an air-cooled He-Ne laser at 532 nm wavelength was focused on the sample surface through a 50×long working distance lens (Olympus America Inc.). The power of laser beam delivered to the sample surface was roughly 10% of the maximum 200 mW laser intensity, unless specified, to avoid degradation to the products. The Raman spectrum acquisition time varied from 100-200 ms with 50 accumulations. At least three different places on the sample surface at each plate were checked to ensure the Raman spectra were credible and reproducible. The spectral resolution of the Raman spectra in the study was ca. 0.2 cm^{-1}.

Electrochemical measurements

Cyclic voltammetry (CV) and linear sweep voltammetry (LSV) with rotating disk electrode (RDE) studies were carried out using a CHI760E electrochemical workstation CH Instruments, Inc. USA) with a 3 electrodes system. For LSV test, a 5 mm diameter rotating glassy carbon disk encased in Teflon was used as the working electrode, a platinum sheet and a sat. Ag/AgCl electrode were used as counter electrode and reference electrode, respectively. For CV test, a 3 mm diameter glassy carbon electrode was used, and the same counter and reference electrodes in LSV test were used. The glassy carbon electrode was polished with alumina slurry (0.05 µm, Al_2O_3 powder) and rinsed with DI water before measurement in both tests. CV curves were acquired at various scan rates of 625, 400, 225, 100, 64, 25, and 9 mV s^{-1} using 2.0 mM active materials in 1.0 M KOH supporting electrolyte. LSV polarization curves were recorded at six typical rotation rates of 100, 225, 400, 900, 1225, 1600, 2025, and 2500 rpm with a sweep rate of 10 mV s^{-1} using the same electrolyte in CV test. CV and LSV were measured after deaerating the electrolyte with N_2 gas. All potentials were calibrated with potassium ferricyanide and converted to the normal hydrogen electrode (NHE).

The redox potential $E_{1/2}$ was calculated as the average of the cathodic and anodic peak potentials. The anodic to cathodic maximum current ratios (I_{pa}/I_{pc}) were plotted

versus the square root of the scan rates. The potential separation ΔE was calculated by $E_{oxidation}$-$E_{reduction}$ at different scan rates. The diffusion coefficients D_0 ($cm^2\ s^{-1}$) of the active species were determined by using the Levich equation:(6,7)

$$i_L=0.620nFAC_0D_0^{2/3}\upsilon^{-1/6}\omega^{1/2}$$

where i_L is the limiting current obtained at a platform voltage (V vs. NHE) from the LSV test, n is the number of electrons involving the redox process (n=2 for first and second redox peaks of DQ-DC to DQ-DC-2H and DQ-DC-4H, n=1 for the third and fourth redox peaks of DQ-DC-5H and DQ-DC-6H; n=2 for first redox peaks of Q-C to Q-C-2H, n=1 for the second redox peak of Q-C-3H; n=1 for first and second redox peaks of FL-DC to FL-DC-H and FL-DC-2H.), F is the Faraday constant (96485 C mol^{-1}), A is the surface area of the working electrode, C_0 is the concentration of the redox-active organic compounds, D_0 is the diffusion coefficient, υ is the kinematic viscosity in $cm^2\ s^{-1}$, and ω is the rotating angular velocity in rad s^{-1}. The current measured at each potential was plotted versus the square root of the electrode rotation rate. The resulting fitted line was expressed by the Koutecký-Levich equation:

$$1/i=1/i_K+1/i_L=1/i_K+1/0.620nFAC_0D_0^{2/3}\upsilon^{-1/6}\omega^{1/2}$$

where i_K is the heterogeneous rate constant. $\log_{10}(i_K)$, as calculated from the vertical-axis intercepts at each potential, was plotted versus the over potential (η), which was calculated from the difference between the measured potential and $E_{1/2}$ for the redox couple. The fitted line was expressed by the Tafel equation:

$$\log_{10}(i)= \log_{10}(i_0)+\alpha nF\eta/RT$$

where i_0 is the exchange current in amp, α is the transfer coefficient, R is the universal gas constant (8.314 J K^{-1} mol^{-1}), and T is temperature (298 K). The exchange current i_0 was determined from the vertical-intercept, and the kinetic rate constant k_0 (cm s^{-1}) was calculated using the equation:

$$k_0=i_0/nFC_0A$$

where the concentrations of the redox couple are meant to be the same.

Battery tests

Battery tests were conducted under a Neware battery test system (CT-4008-5V6A-S1-

F, Neware, Shenzhen, China). A lab-made full battery was assembled with two graphite end plates, two Cu current collectors, two graphite felt, and a piece of CEM as the separator. The active electrode and membrane area was 5.0 cm^2. During operation, both the anolyte and catholyte were flowed through the RFB at a constant flow rate of 60 mL min^{-1} using a peristaltic pump to ensure uniform mass transport and minimize concentration polarization.

Three configurations of the ROM-based RFBs were primarily explored: the first one was 0.025 M solutions of DQ-DC, Q-C, or FL-DC in 1.0 M KOH (7.0 mL) were employed as the anolytes, with $K_4Fe(CN)_6$ in 1.0 M KOH serving as the catholyte. In the second and third configurations, DQ-DC was used at higher concentrations of 0.1 M and 0.5 M, respectively, under the same alkaline conditions. In all cases, the capacity of the catholyte was maintained at three times that of the anolyte to ensure excess electron acceptor availability.

Galvanostatic charge-discharge tests were carried out under the following conditions: For the 0.025 M DQ-DC, Q-C, and FL-DC systems, cycling was conducted at a current density of 20 mA cm^{-2}, with cutoff voltages set at 1.5-1.6 V for the charging process and 0.4-0.5 V for discharging, respectively. For the 0.1 M and 0.5 M DQ-DC systems, current densities of 30 and 50 mA cm^{-2} were applied, respectively, with uniform cutoff voltages of 1.55 V for charging and 0.50 V for discharging.

To assess rate performance, the 0.1 M DQ-DC RFB was subjected to galvanostatic cycling at a range of current densities from 10 to 90 mA cm^{-2} in increments of 10 mA cm^{-2}, using the same voltage window. All electrochemical measurements were conducted in an Ar-filled glovebox at room temperature (~25 °C) to ensure an inert and controlled testing environment.

Density functional theory calculations

All density functional theory (DFT) calculations were performed with the Gaussian 16. The geometry optimizations and frequency calculations were performed using B3LYP functional with 6-311+G(d,p) basis set(8,9). The D3 dispersion correction was added to ensure proper description of dispersive interactions. The implicit SMD solvent model

was used to represent the solvent effect of water molecule(10,11). Atomic dipole corrected Hirshfeld atomic charge (ADCH) and electron-density analysis were undertaken with the Multiwfn program (12-14).

Nucleus-independent chemical shifts (NICS) were calculated at the same level according to the procedure described by Jonathan R. Nitschke (15). NICS(1)zz is the negative value of the shielding tensor component perpendicular to the ring plane at 1 angstrom above the ring plane (specifically, by moving 0.1 nm perpendicular to the ring plane from the center of the ring). Anisotropy of the induced current density (ACID) Plots (B3LYP/6-31G(d)) was calculated using the method developed by Herges.(16) The result visualization was carried out by using POV-Ray v3.7 software.

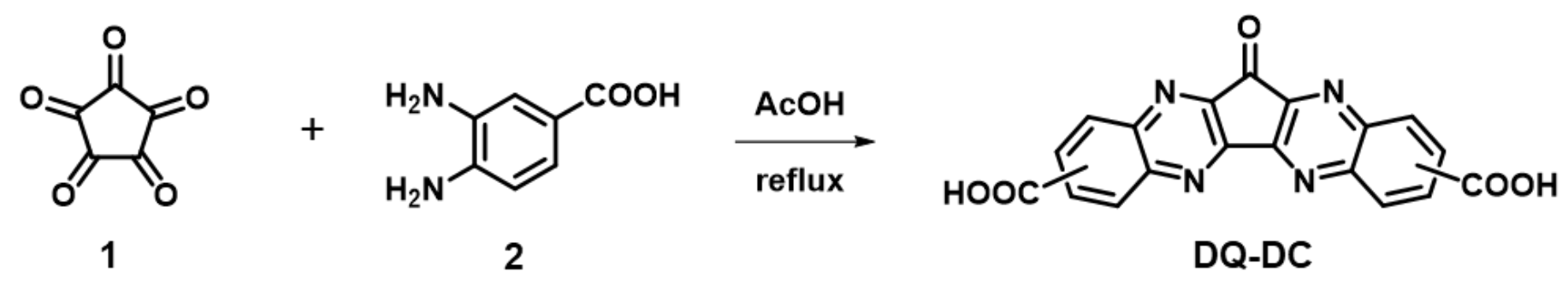

Figure S1. Synthetic route illustrations for **DQ-DC**.

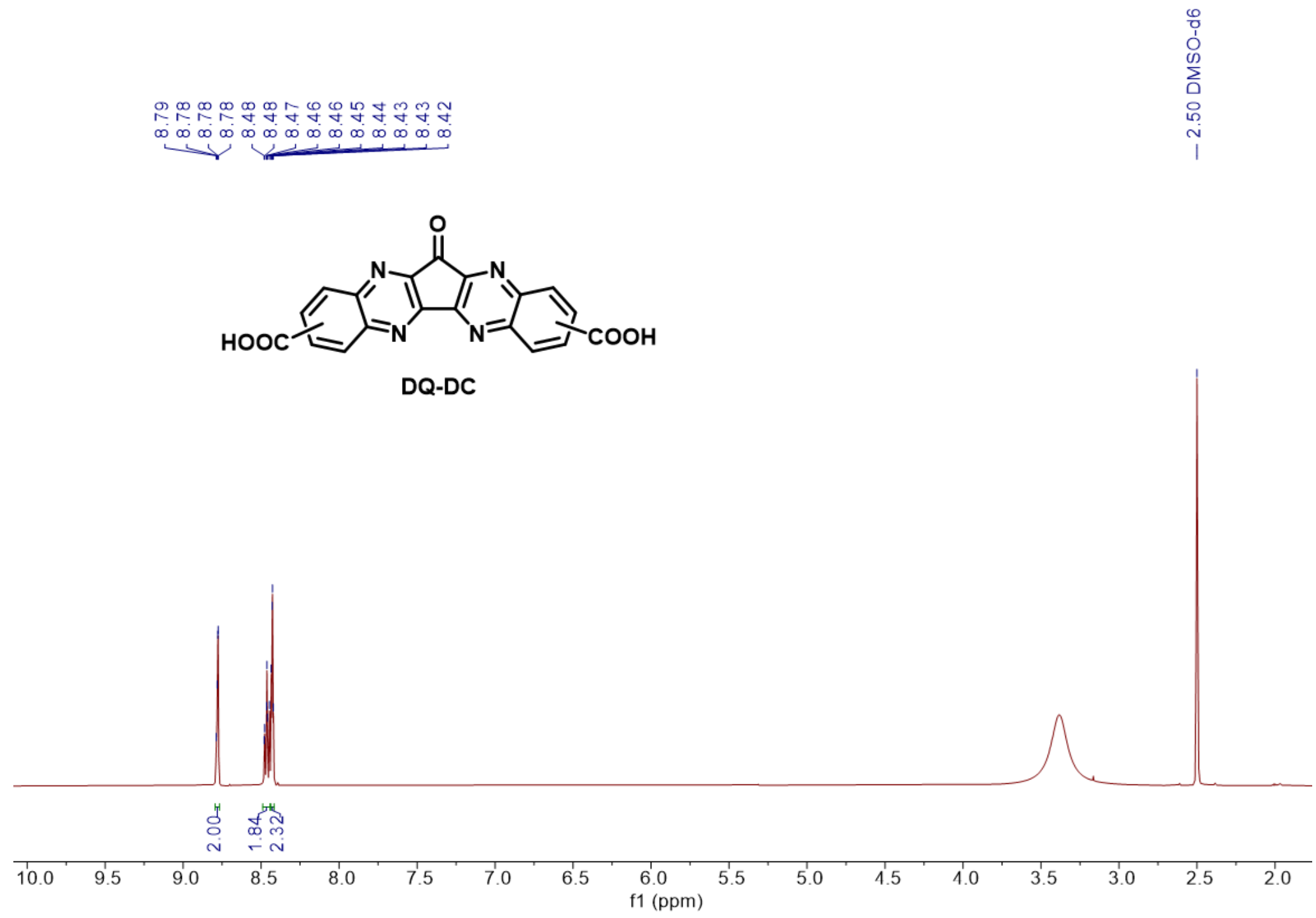

Figure S2. ^{1}H NMR spectrum of **DQ-DC**, recorded in DMSO-d_6.

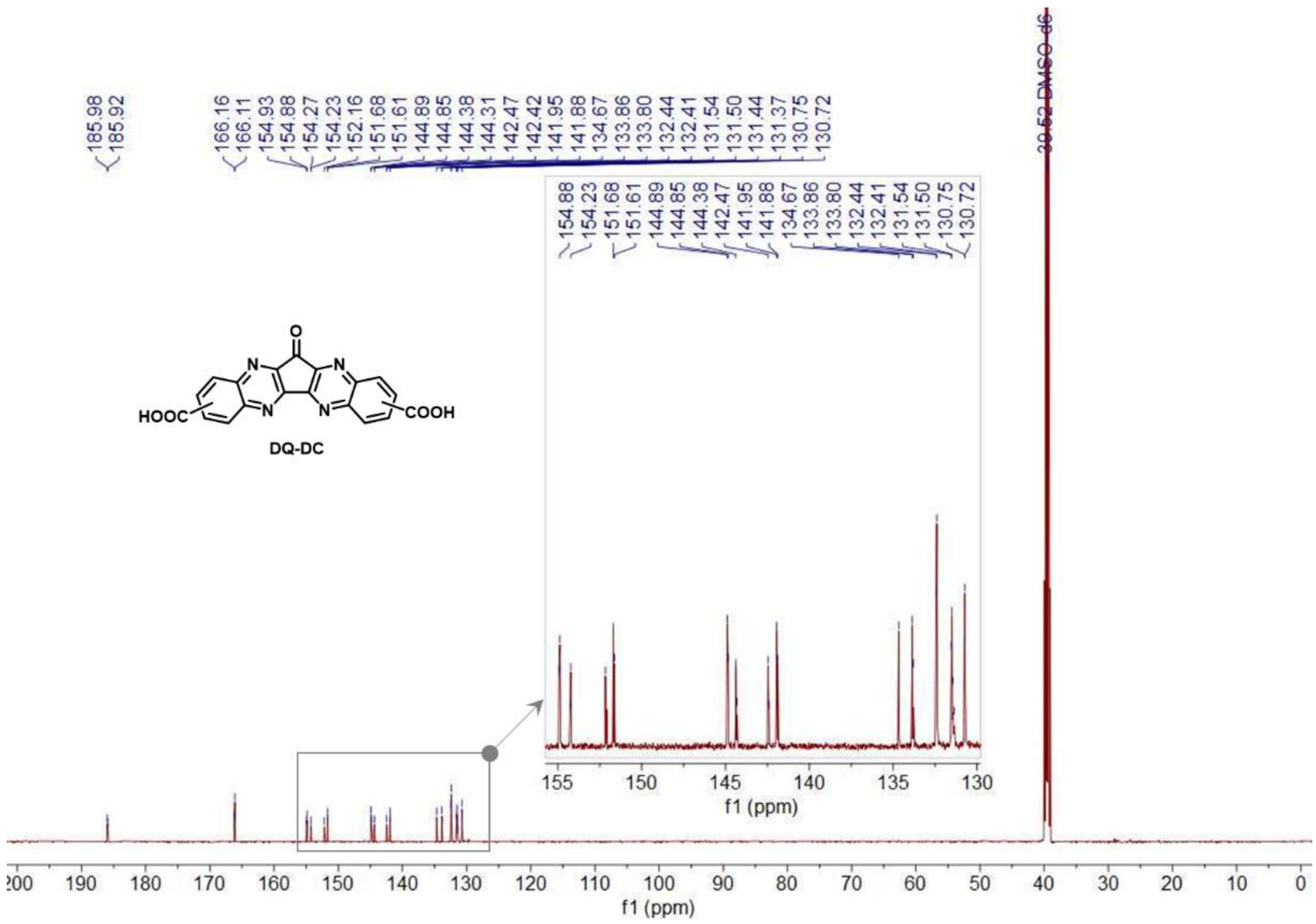

Figure S3. ^{13}C NMR spectrum of **DQ-DC**, recorded in DMSO-d_6. Inset is the partially enlarged spectrum. The inconsistency in carbon numbers suggests the possible presence of isomers.

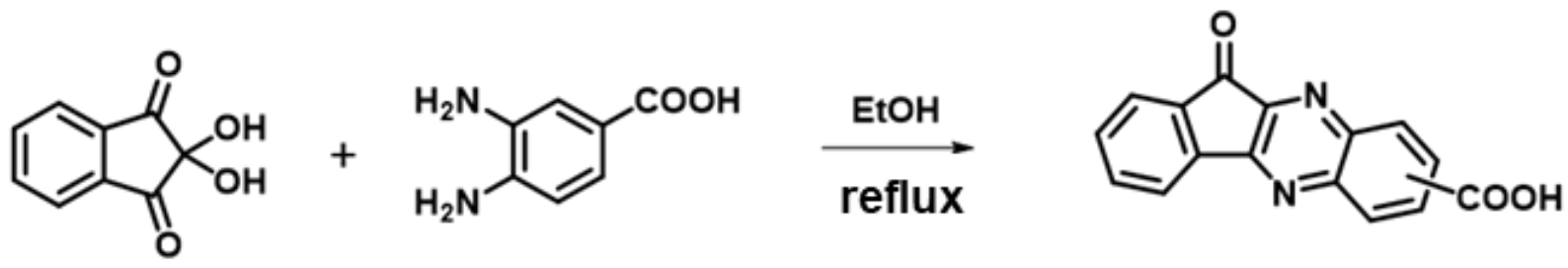

3 2 Q-C

Figure S4. Synthetic route of **Q-C**.

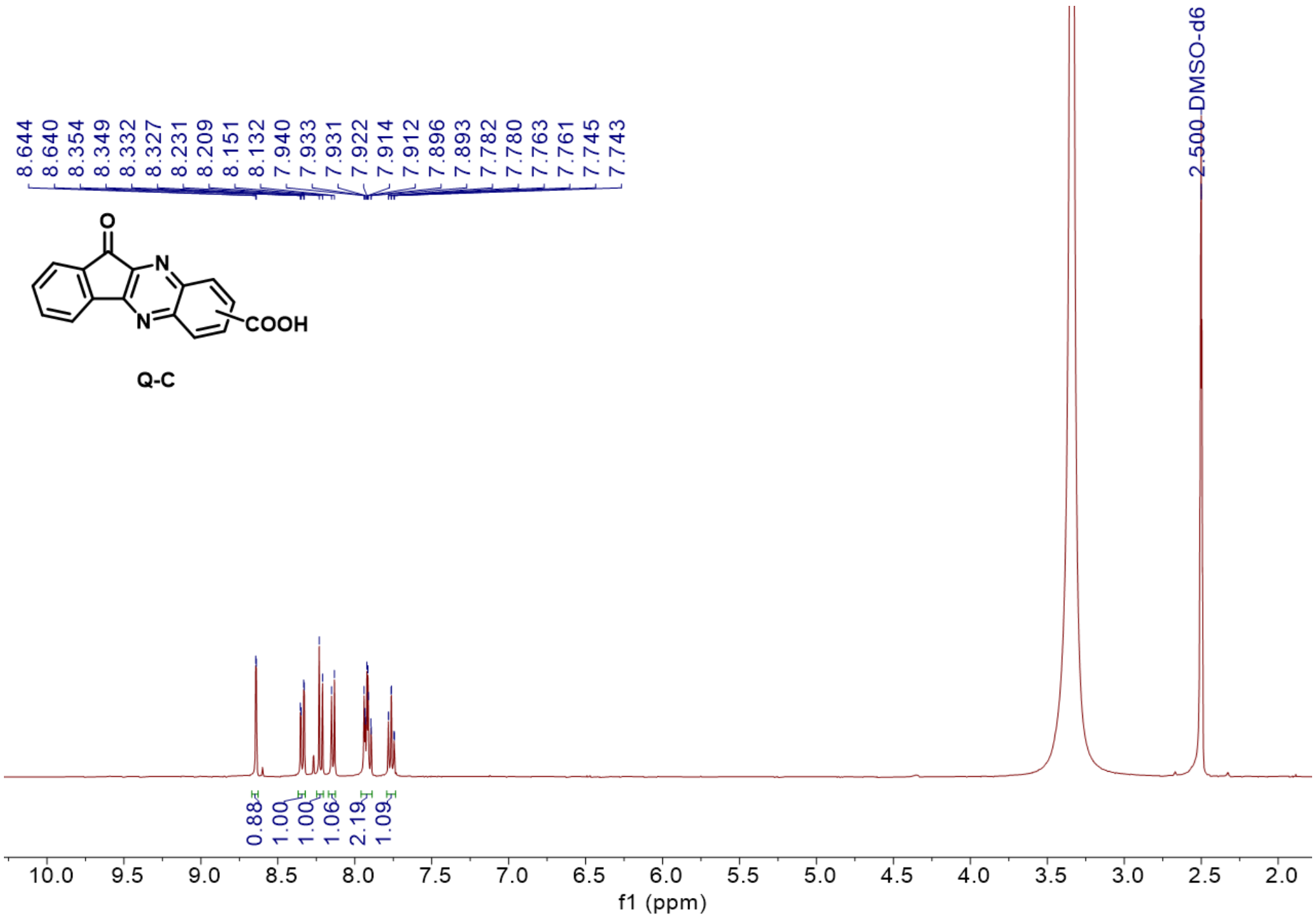

Figure S5. ^{1}H NMR spectrum of **Q-C**, recorded in DMSO-d$_6$.

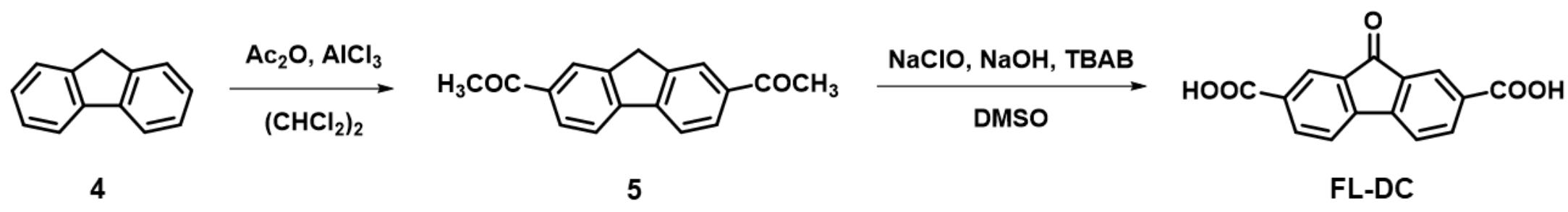

Figure S6. Synthetic route of **FL-DC**.

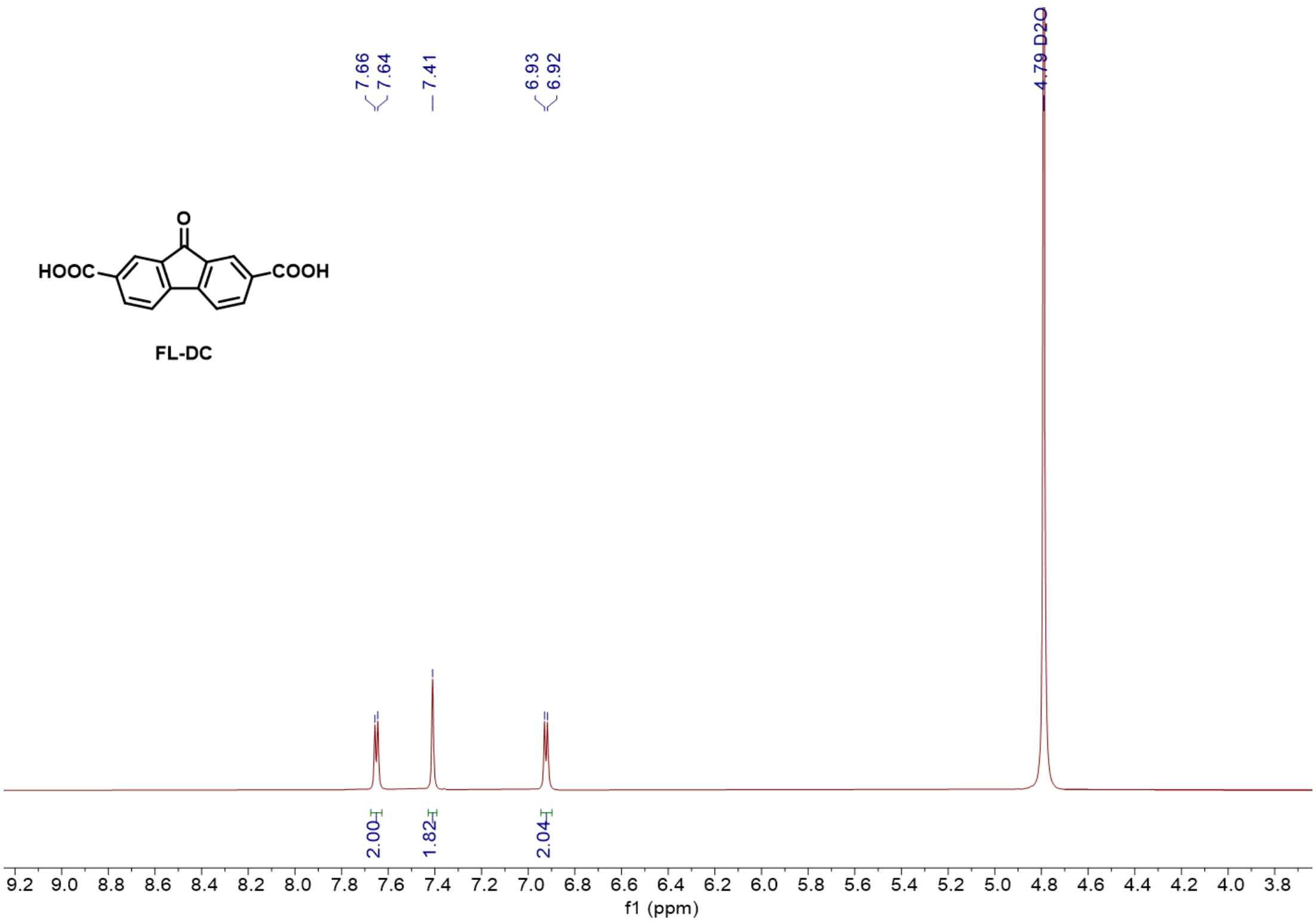

Figure S7. ^{1}H NMR spectrum of **FL-DC**, recorded in D_2O.

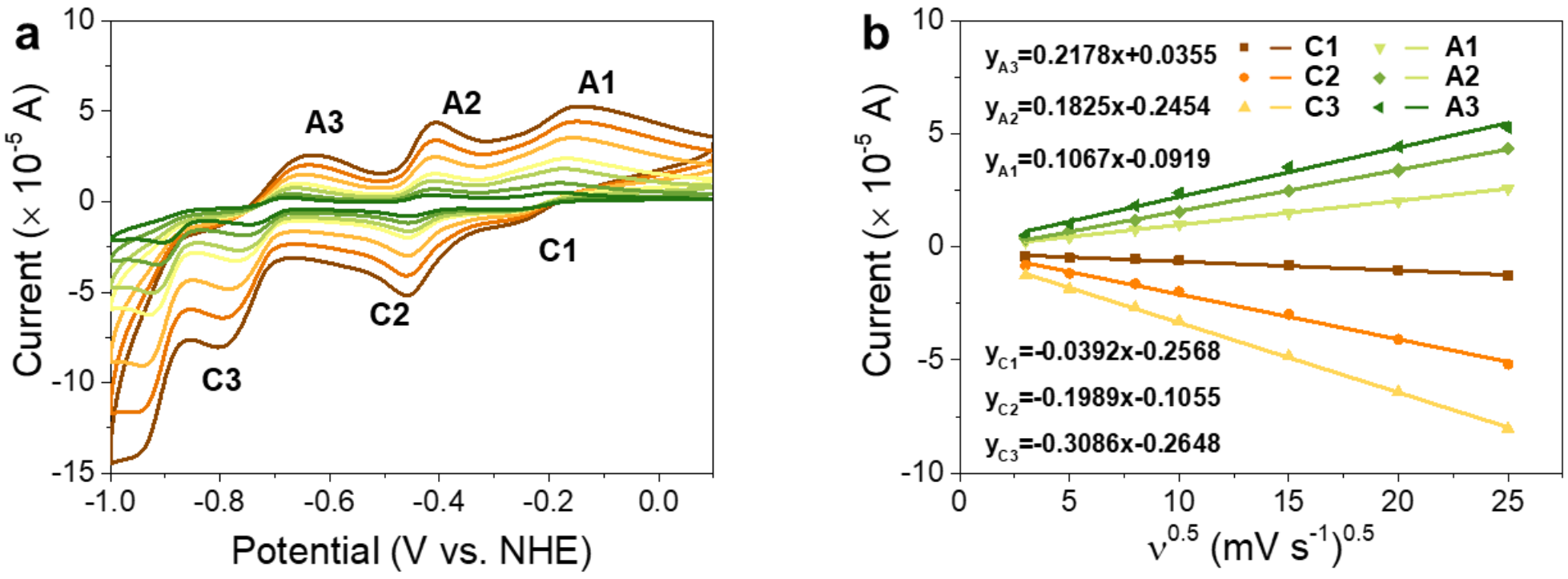

Figure S8. CV analyses for **DQ-DC** in 1.0 M KOH. (a) CV curves recorded at scan rates range from 9 to 625 mV s^{-1}. (b) Linear relationship between the peak current values and the square root of the scan rate $\nu^{0.5}$ diagram. DQ-DC concentration: 4 mM.

The Pourbaix diagram demonstrates significant pH dependence of the redox potential, consistent with proton-coupled electron transfer behavior (**Figure S9**). At pH values above ~10, DQ-DC exhibits two sequential $2H^+/2e^-$ redox processes, which correspond to strongly proton-coupled electron transfer pathways, as reflected by slopes near -60 mV per pH unit. At the same time, a $1H^+/2e^-$ process is also observed, which is indicative of weaker proton coupling, with slopes approaching -34 mV per pH unit. Additionally, at pH values below ~10, a redox process with little to no associated proton transfer is evident, consistent with a zero-proton, multi-electron redox event.

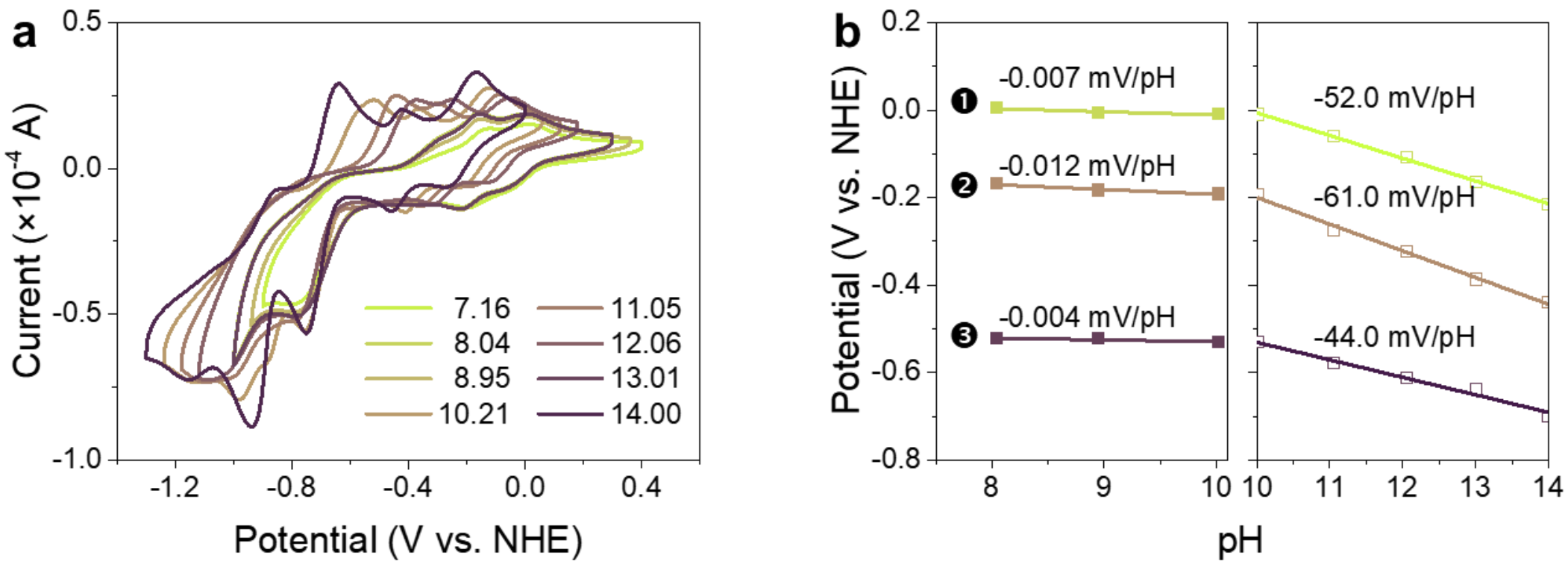

Figure S9. Redox properties of DQ-DC. (a) CV curves of DQ-DC measured in solutions with different pH values at 100 $mV\cdot s^{-1}$. (b) The corresponding Pourbaix diagrams.

Semi-in situ ^{1}H NMR was performed in tandem with EPR and Raman characterizations at defined stages of the charging process (**Figures S9-S11**). At the onset of charging, the NMR spectrum shows a prominent proton signal corresponding to the aromatic protons of DQ-DC (**Figure S9**). As charging progresses from positions ① to ⑥, the intensity of the aromatic proton signal in the 8.0-9.0 ppm region gradually decreases. This trend is consistent with the EPR data between 3480 and 3510 Gaussian (**Figure S9**), which reveal the generation of significant radical signals during the reduction of the phenazine units. Notably, the EPR radical intensity exhibits two distinct increases followed by decreases, which correlates with the sequential reduction of the two phenazine moieties in DQ-DC.

During subsequent stages from positions ⑦ to ⑬, the ^{1}H NMR spectra exhibit a downfield shift of aromatic proton resonances into the 6.0-7.5 ppm region, consistent with increased electron density in the reduced molecular states. Concurrently, new resonances appear in the 3.0-4.5 ppm region, which we attribute to C-H protons formed in the alcohol group after reduction of the fluorenone C=O moiety. These observations provide direct evidence for the stepwise redox sequence in which the phenazine units are reduced first, followed by reduction at the fluorenone carbonyl. EPR data throughout this process also reveals transient, low-intensity radical signals in later reduction stages (**Figure S10**), which may contribute to additional structural features observable in the NMR spectra but are not readily resolved into precise integrals.

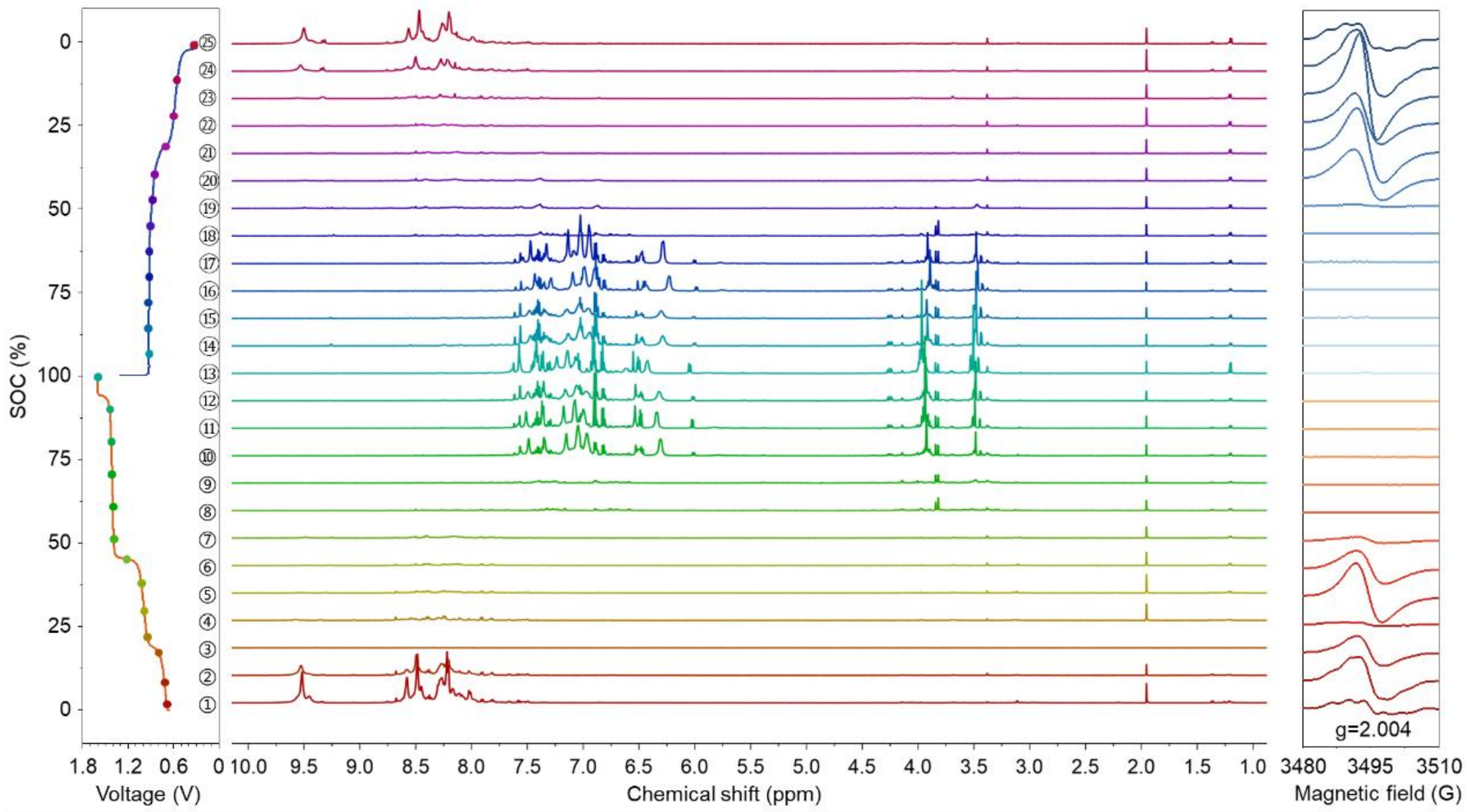

Figure S10. First-cycle electrochemical and spectroscopic characterizations of the DQ-DC-based battery. Galvanostatic-potentiostatic charge-discharge profile of the first cycle, corresponding semi-in-situ ^{1}H NMR spectra, and the associated EPR spectra recorded during the same cycle. NMR measurements were conducted with D_2O as the solvent, and the reported EPR signal corresponds to a g-factor of 2.004.

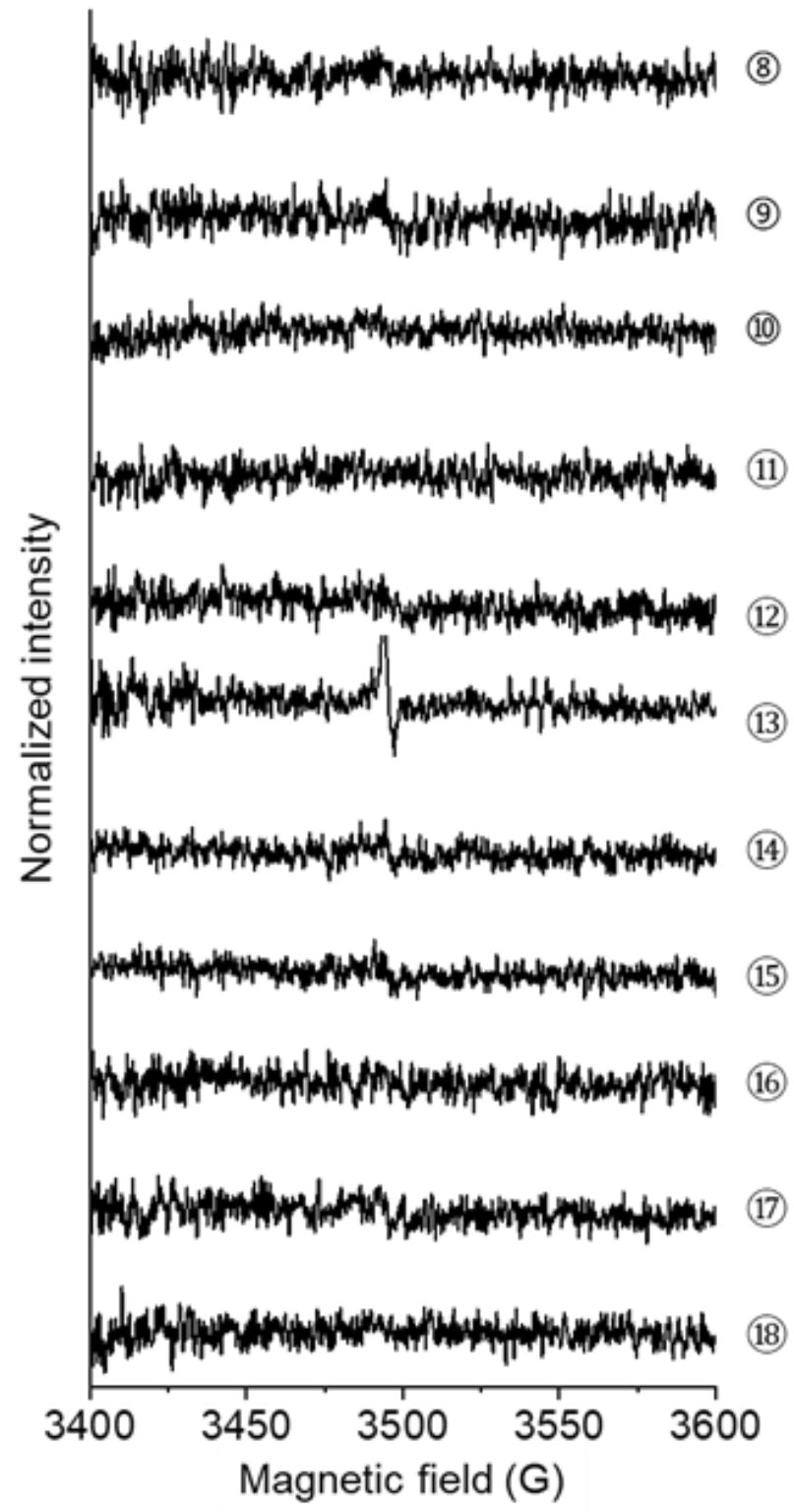

Figure S11. Magnified semi-in-situ EPR spectra (spectra ⑧–⑱). Enlarged view of the semi-in-situ EPR signals corresponding to spectra numbered ⑧ to ⑱.

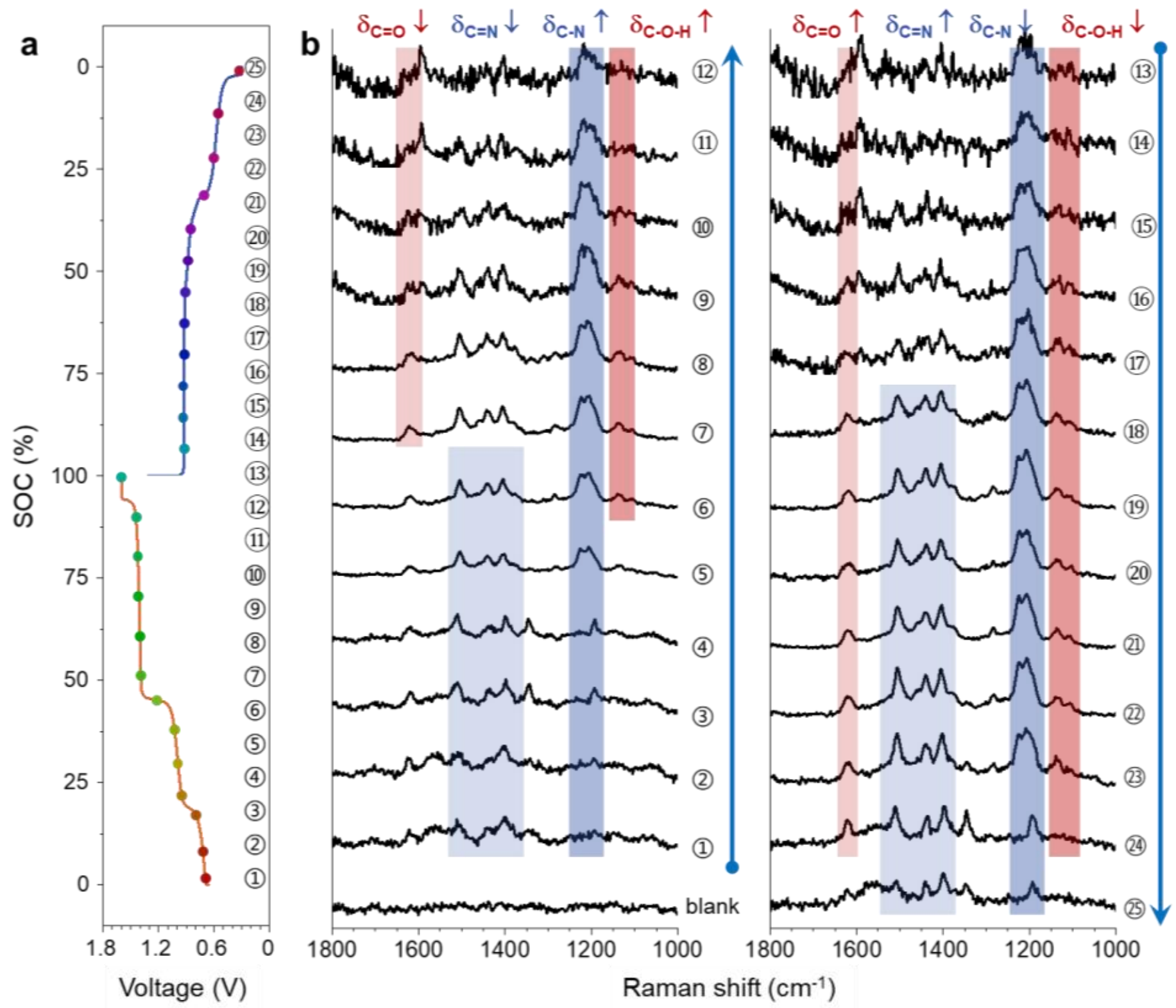

Figure S12. First-cycle galvanostatic-potentiostatic charge-discharge profile and semi-in-situ Raman analysis of DQ-DC-based battery.

Figure S11 summarizes the evolution of Raman vibrational features during the charging and discharging. During charging, the C=N stretching vibration of the aromatic heterocycle in the ~1500-1650 cm^{-1} region gradually decreases, consistent with a change in electronic structure associated with reduction of the heterocyclic core. In contrast, we observe a progressive increase in the intensity of the C-N stretching band in the ~1100-1350 cm^{-1} region, indicative of changes in the local environment of the phenazine moiety.

Importantly, the C=O stretching band of the fluorenone core in the ~1650-1750 cm^{-1} region remains largely unchanged at early stages of charging, but begins to diminish in intensity from around the seventh measured point onward. At the same time, we observe a corresponding increase in the intensity of the C-O stretching bands in the ~1000-1200 cm^{-1} region, which are attributed to the formation of C-OH species as the carbonyl group undergoes reduction. These FTIR changes are consistent with sequential reduction behavior in which the phenazine units are reduced prior to the fluorenone C=O group, supporting the stepwise multi-electron transfer hypothesis.

During the discharge process, the observed Raman bands exhibit the reverse pattern. The intensity of the C-O band decreases and the C=O band recovers, while the C=N and C-N features move back toward their original states.

Taken together, the NMR, EPR, and Raman data sets provide a coherent picture of the multi-step redox process, with specific functional groups undergoing predictable and reversible transformations consistent with the proposed six-electron transfer mechanism.

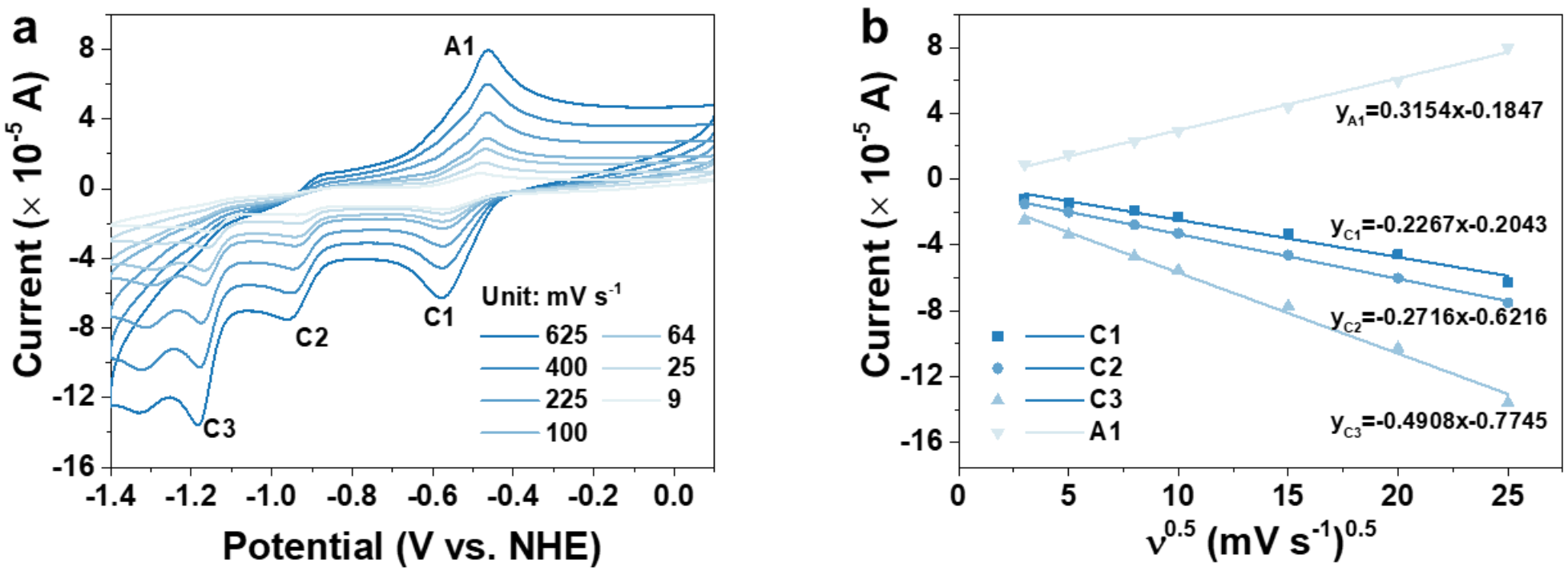

Figure S13. CV analyses for **Q-C** in 1.0 M KOH. (a) CV curves recorded at scan rates range from 9 to 625 mV s^{-1}. (b) Linear relationship between the peak current values and the square root of the scan rate $\nu^{0.5}$ diagram. Q-C concentration: 4 mM.

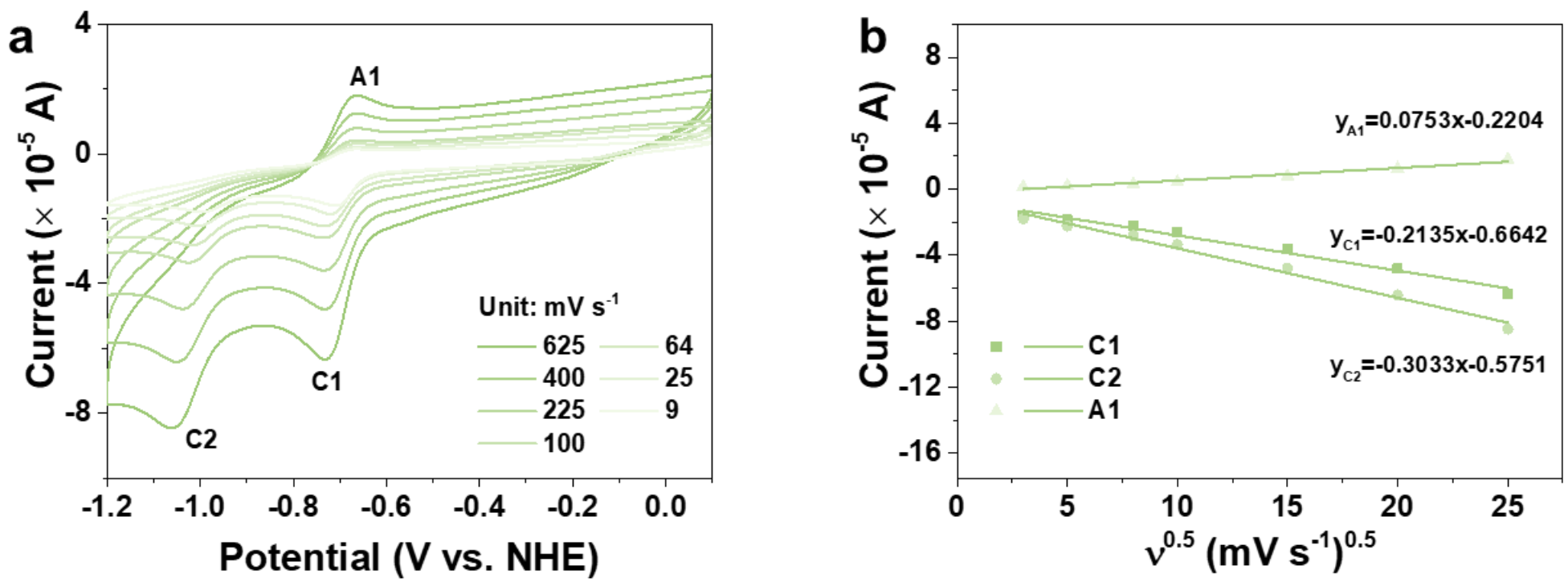

Figure S14. CV analyses for **FL-DC** in 1.0 M KOH. CV curves recorded at scan rates range from 9 to 625 mV s^{-1}. (b) Linear relationship between the peak current values and the square root of the scan rate $\nu^{0.5}$ diagram. FL-DC concentration: 4 mM.

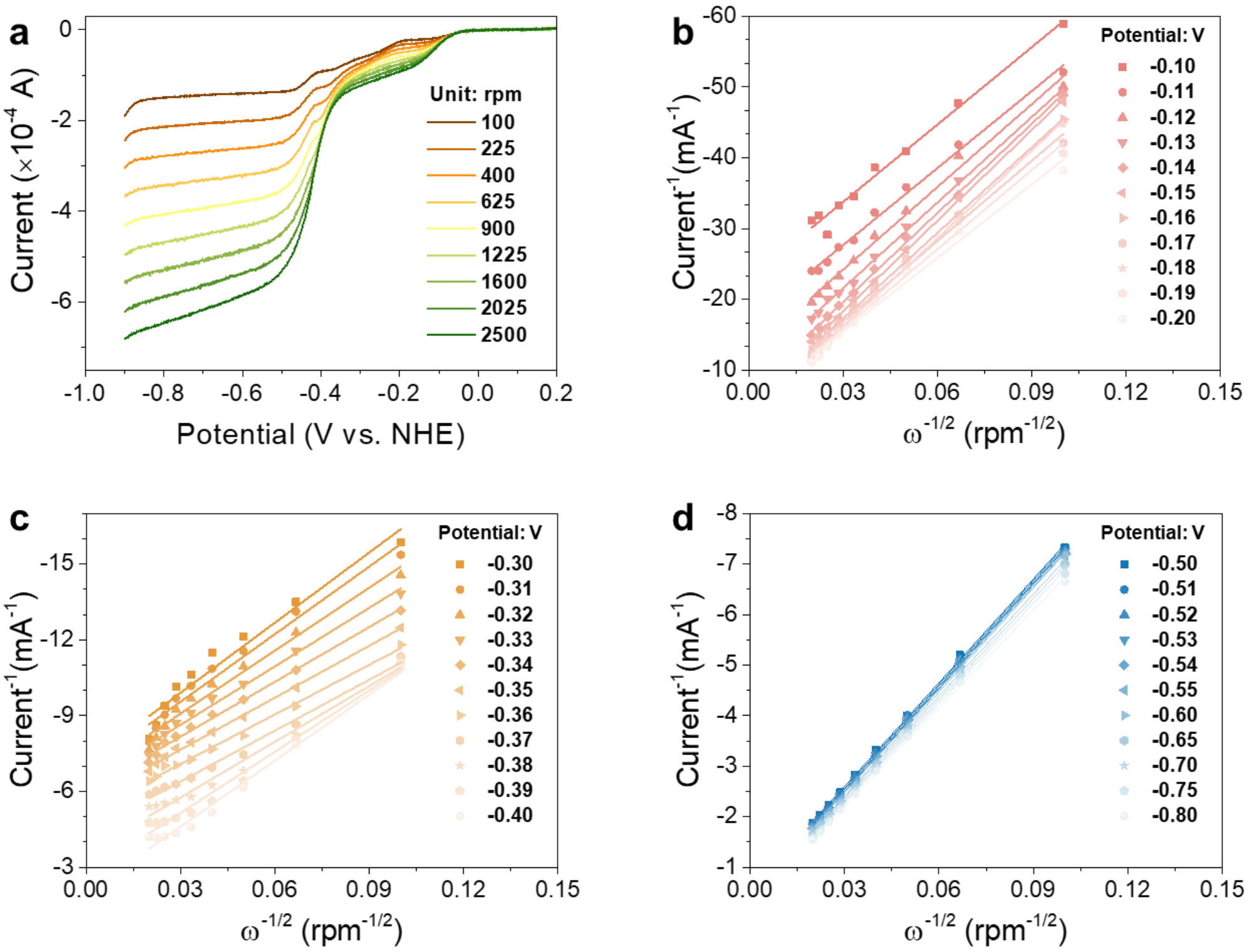

Figure S15. LSV analyses for **DQ-DC** in 1.0 M KOH. (a) LSV curves recorded at various rotation rates range from 100 to 2500 rpm. (b-d) Koutecký-Levich curves (current^{-1} vs $\omega^{-1/2}$) at different reduction overpotentials. DQ-DC concentration: 4 mM. Sweep rate: 10 mV s^{-1}.

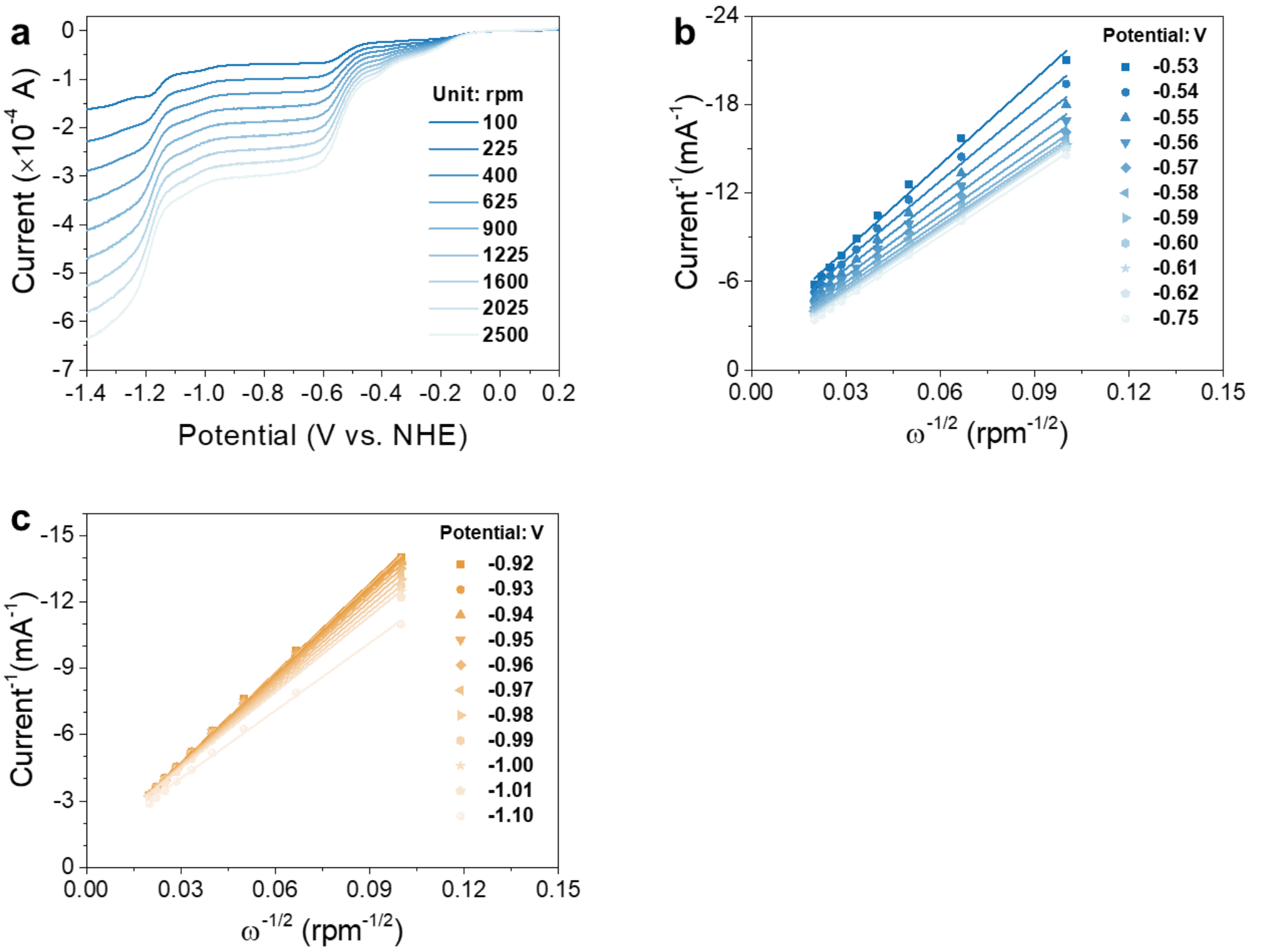

Figure S16. LSV analyses for **Q-C** in 1.0 M KOH. (a) LSV curves recorded at various rotation rates range from 100 to 2500 rpm. (b-c) Koutecký-Levich curves (current^{-1} vs $\omega^{-1/2}$) at different reduction overpotentials. Q-C concentration: 4 mM. Sweep rate: 10 mV s^{-1}.

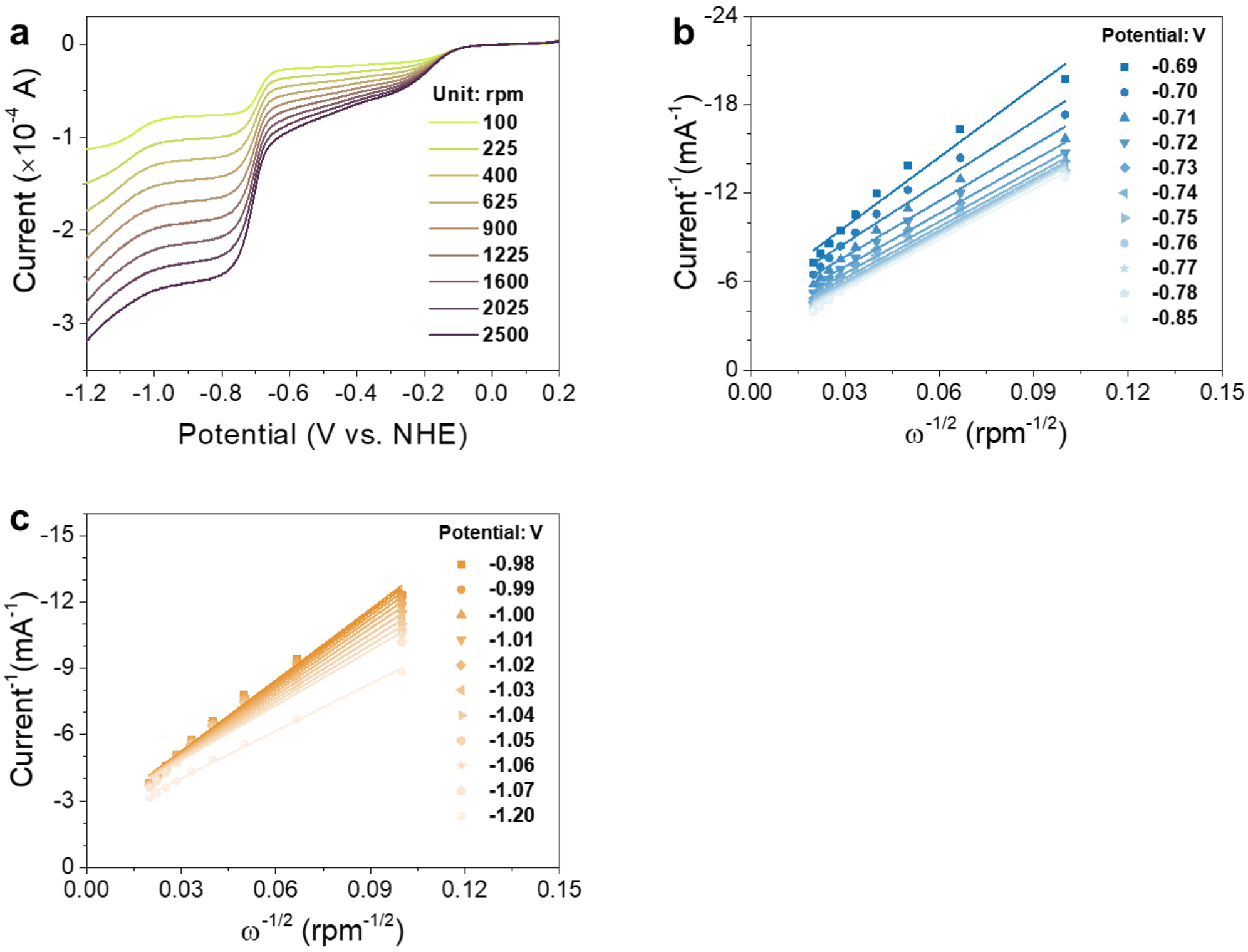

Figure S17. LSV analyses for **FL-DC** in 1.0 M KOH. (a) LSV curves recorded at various rotation rates range from 100 to 2500 rpm. (b-c) Koutecký-Levich curves (current^{-1} vs $\omega^{-1/2}$) at different reduction overpotentials. FL-DC concentration: 4 mM. Sweep rate: 10 mV s^{-1}.

Table S1. The comparison of mass- and charge-transfer-controlled kinetic parameters of DQ-DC and previously reported redox-active materials in RFBs.

Abbreviations	D_o (×10^{-5} cm^2 s^{-1})	k_o (×10^{-3} cm s^{-1})	Refs
**DQ-DC**	**3.81**<br>**1.40**<br>**0.22**	**36.6**<br>**2.65**<br>**1.36**	**This study**
**Q-C**	**1.53**<br>**1.28**	**31.8**<br>**7.64**	
**FL-DC**	**1.05**<br>**0.88**	**20.6**<br>**7.66**	
2,7-AQDS	0.38	7.25	(17)
PTO-PTS	N.A.	29.6<br>31.6<br>17.9	(18)
Cys-DHAQ	1.03	0.88	(19)
DHAQDS	0.32	15.6	(20)
$(CBu)_2NDI$	0.35<br>0.14	10.3<br>35.8	(21)
NDI	0.14-0.20	0.37~0.51; 13.9~17.3	(22)
IC-H	2.19	0.19	(23)
NR	1.70	27	(24)
NHPI	0.54-0.74	12.7~15.9	(25)
MV	2.57	0.30	(26)
$(SO_3)V(OH)Br$	0.63-1.15	1.96~3.91	(27)
$(bpy-(CH_2)_3)NMe_3)I_2$	0.55~2.45	0.39~30.4	(28)
$((NPr)_2TTz)Cl_4$	31.5	0.28	(29)
R-Vi	0.70	0.17	(30)
VIOTEMP	29	58	(31)
BTMAP-Fc	0.31	14	(32)
4-HO-TEMPO	2.95	2.6	(33)
CPL	0.70	6.4	(34)

Table S2. Quantitative comparison of key molecular parameters for representative redox-active organic molecules.

Anolytes	Electron-transfer number	Redox potential	Maximum solubility	Ref.
**DQ-DC**	**6e**	**-0.204 V, -0.418 V, -0.707 V, -0.872 V vs. NHE**	0.57 M (1 M KOH)	**This study**
$AQDS(NH_4)_2$	$2e^-$	-0.20 V vs. NHE	1.3 M (1.0 M NH_4I)	(35)
4C7SFL	$2e^-$		1.5 M (2 M NaOH)	(36)
1,6-DPAP	$2e^-$	-0.56 V vs. SHE	1.005 M (H_2O)	(37)
DHPS	$2e^-$	-1.06 V vs. Ag/AgCl	1.45 M (2 M NaOH)	(38)
BHPC	$2e^-$	-0.78 V	1.55 M (1 M KOH)	(39)
1,8-PFP	$2e^-$	-0.588 V vs. SHE	1.46 M (pH 14)	(40)
$(TPyTz)Cl_6$	$4e^-$	-0.25 V, -0.44 V, -0.63 V vs. SHE	0.30 M (1.2 M e^-)	(41)
ACA	$2e^-$	-0.62 V vs. SHE	2 M (pH 14)	(42)
HATNTA	$6e^-$	-0.49 V, -0.67 V, -0.74 V vs. NHE	0.583 (1.5 M KOH)	(43)

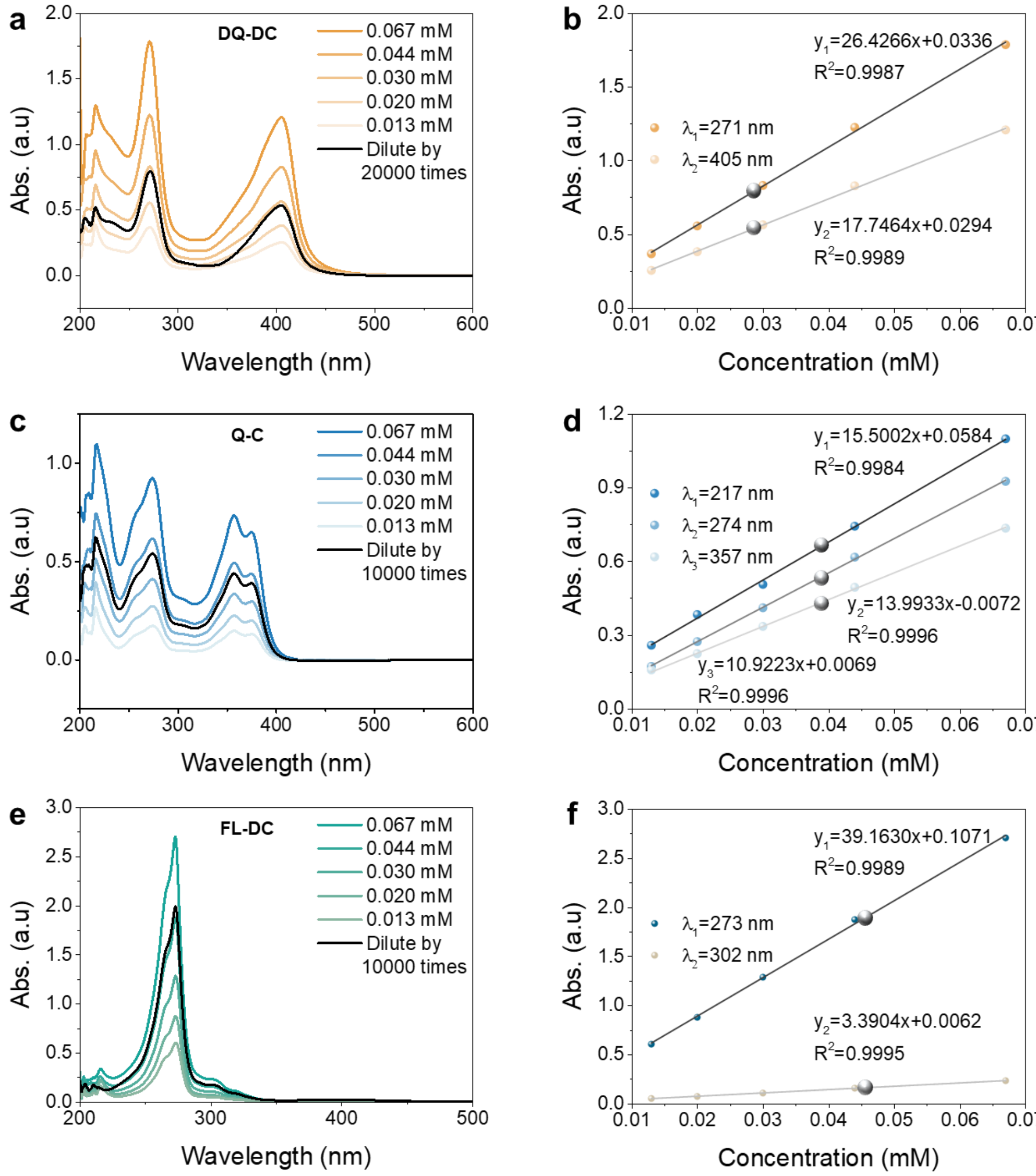

Figure S18. UV-Vis spectra used to determine the aqueous solubility of DQ-DC, Q-C, and FL-DC. (a, c, e) UV-Vis absorption spectra of DQ-DC, Q-C, and FL-DC at varying concentrations in 1.0 M KOH. (b, d, f) Calibration plots showing the absorbance at the respective maximum absorption wavelength as a function of concentration.

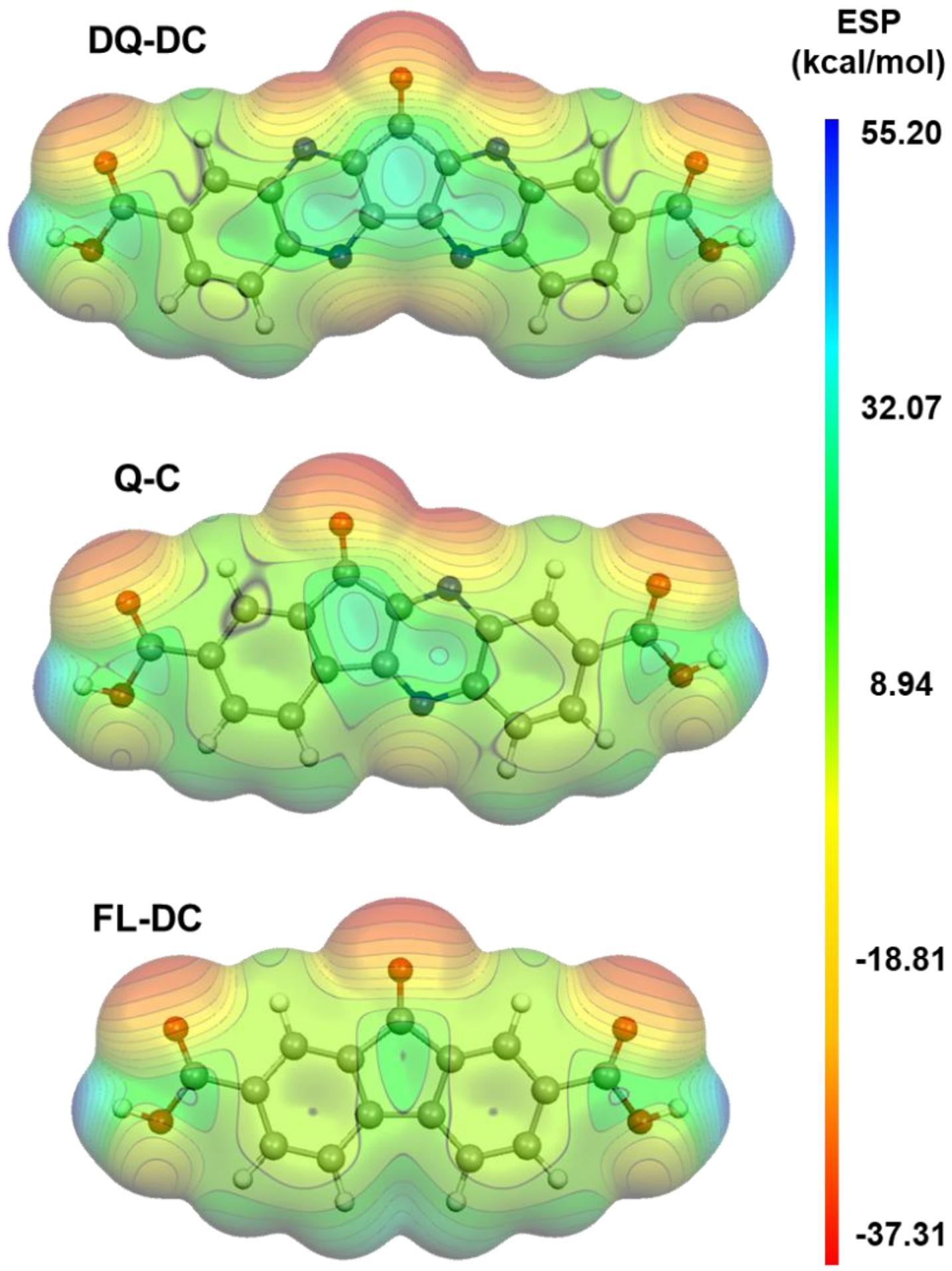

Figure S19. The ESP interpenetration diagrams of DQ-DC, Q-C, and FL-DC.

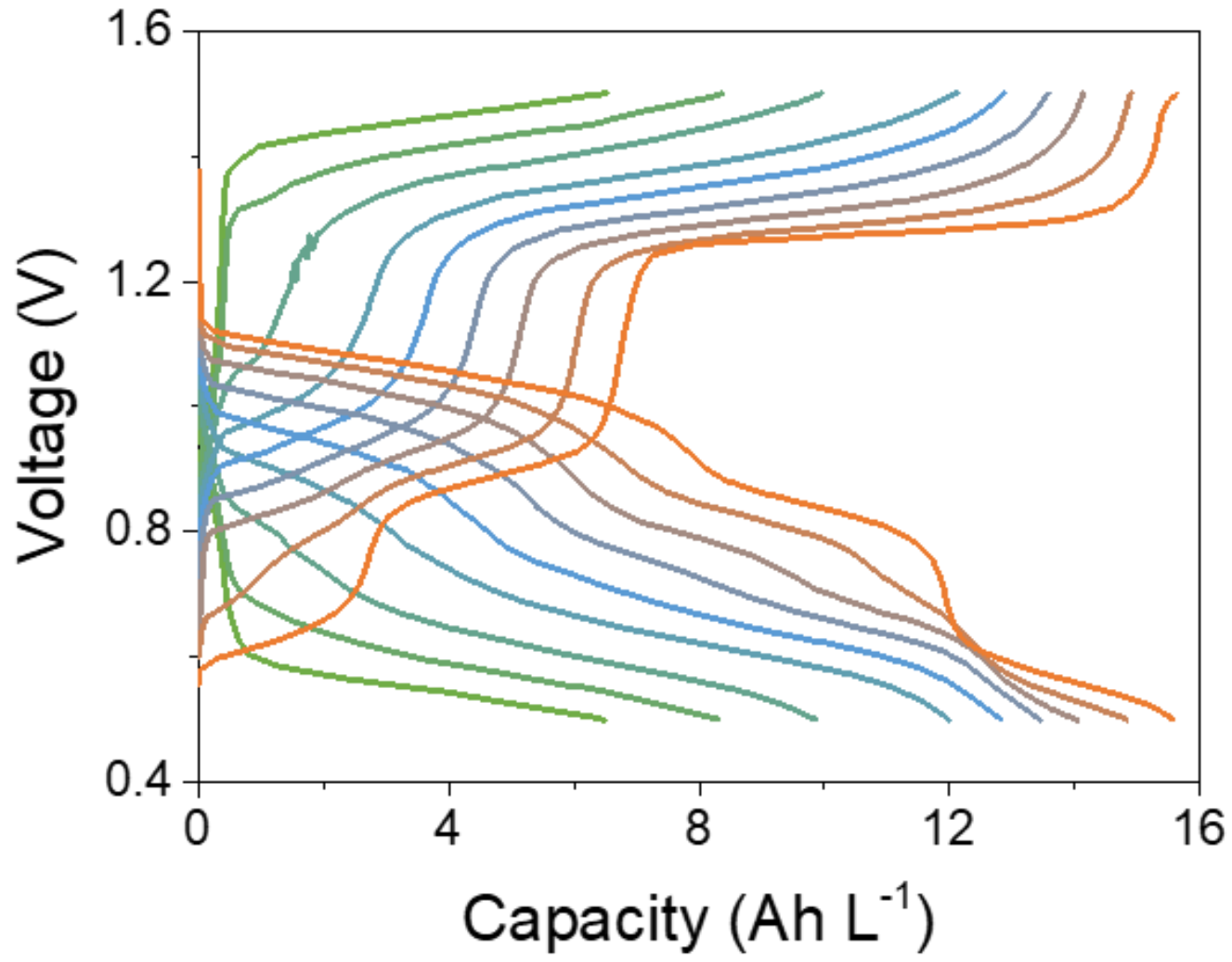

Figure S20. Representative charge-discharge voltage profiles of the 0.1 M DQ-DC-based RFB during galvanostatic cycling at 10, 20, 30, 40, 50, 60, 70, 80, and 90 mA cm^{-2}.

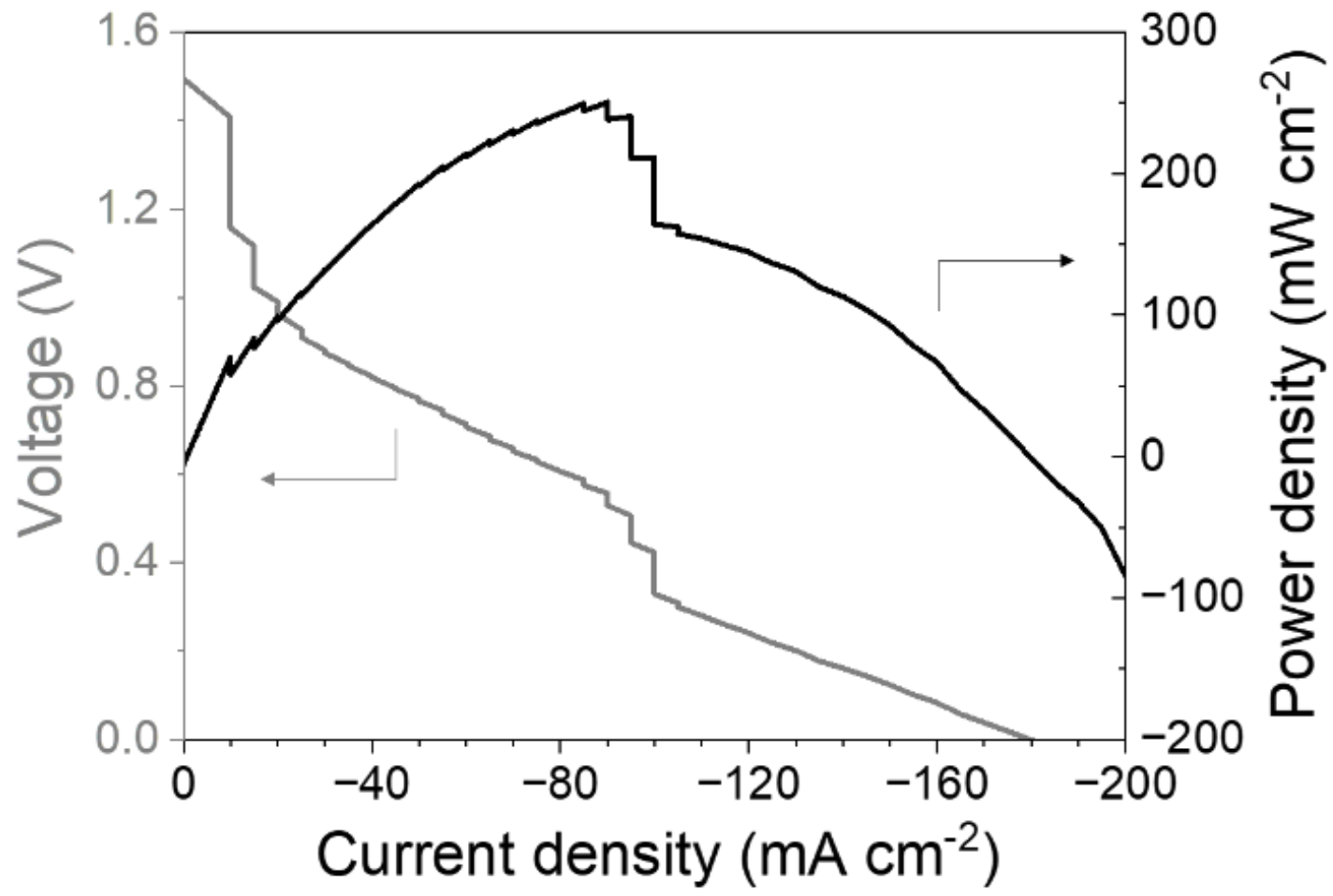

Figure S21. Voltage-current and power density curves of DQ-DC.

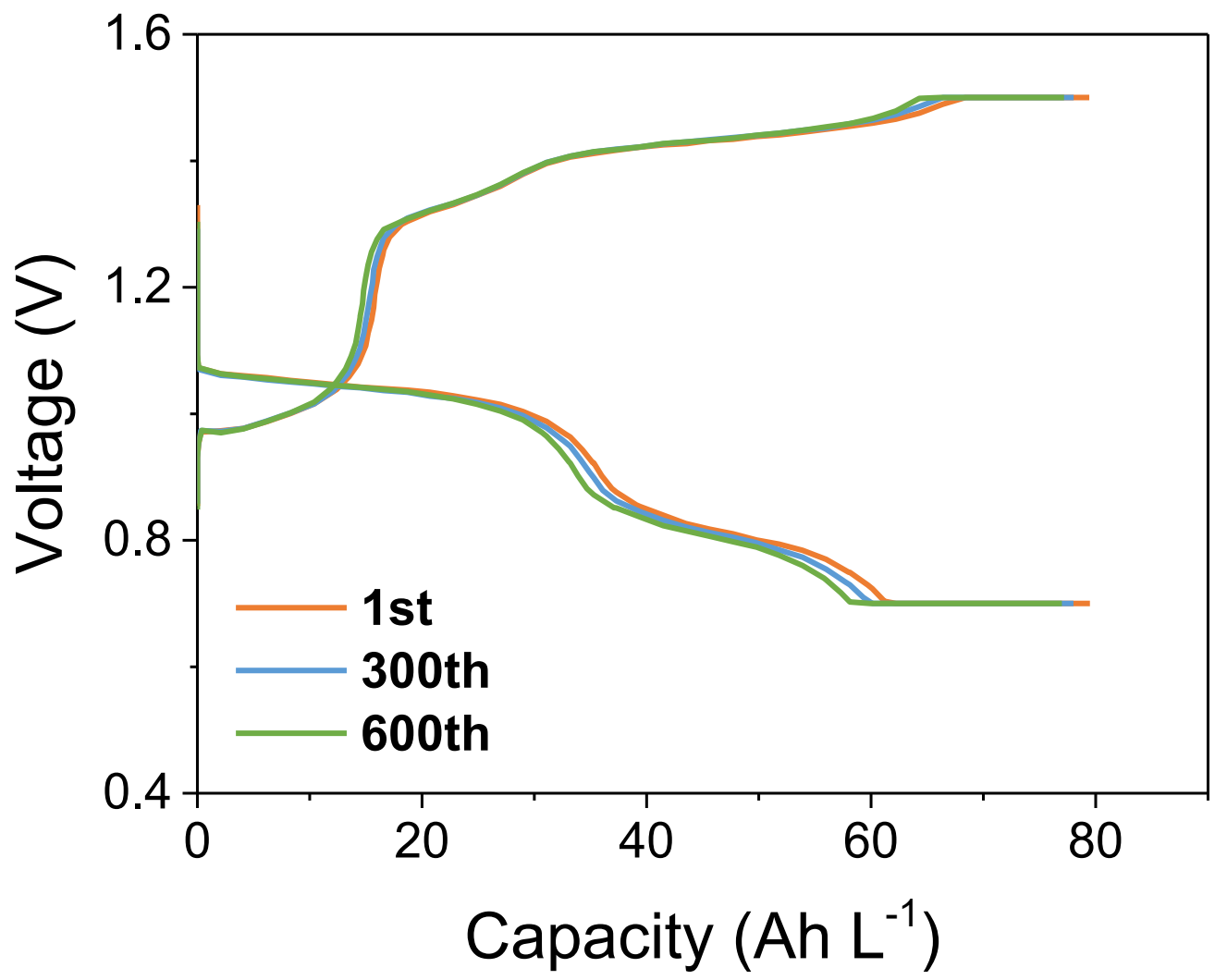

Figure S22. Representative battery voltage versus charge-discharge capacity curves of the 0.5 M DQ-DC-based RFB during galvanostatic-potentiostatic cycling at 1st, 300th, and 600th. The battery was galvanostatically conducted at 50 mA cm^{-2} and each half-cycle ended with a potentiostatic hold until the magnitude of the current density fell below 2.0 mA cm^{-2}.

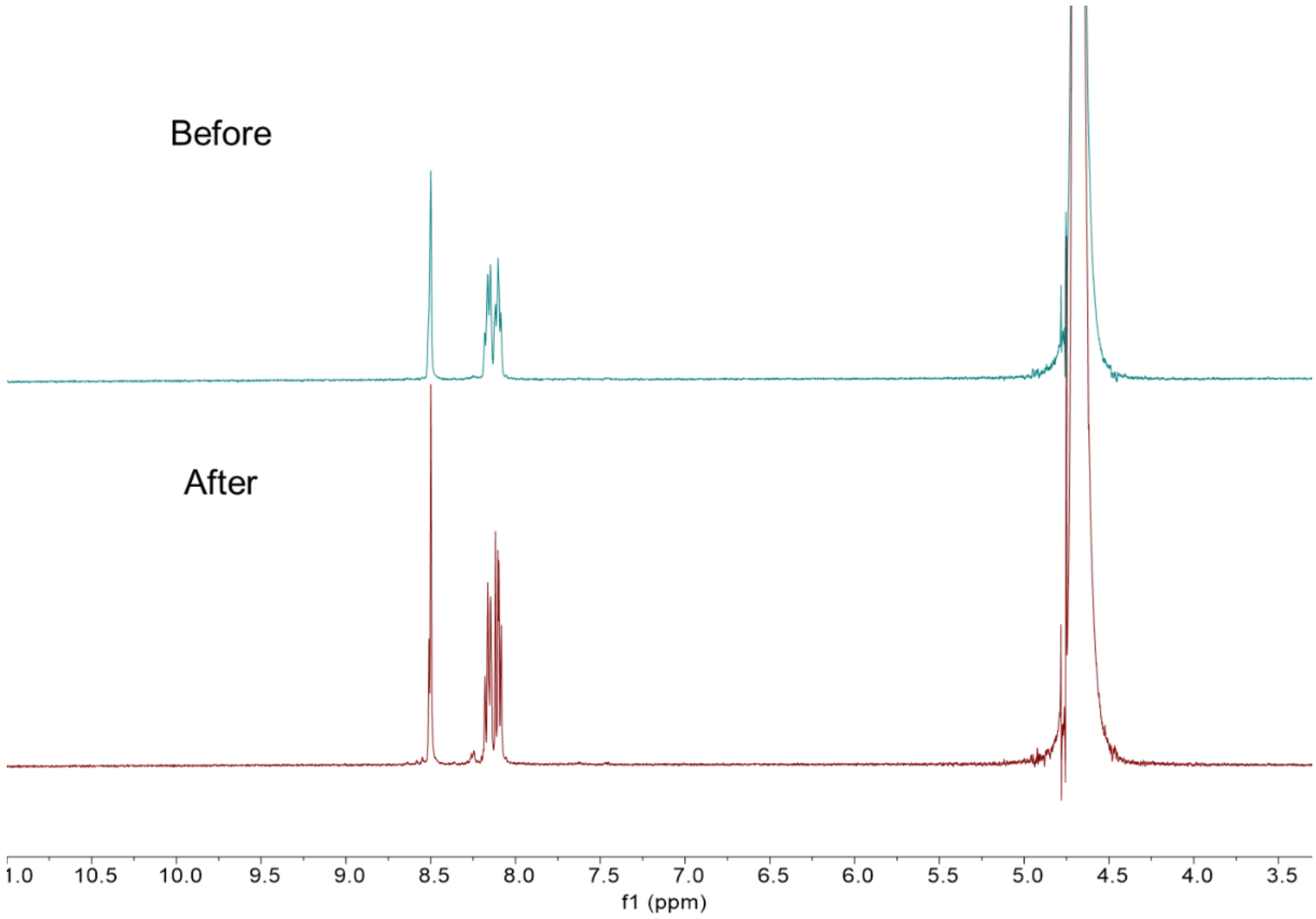

Figure S23. Post-cycling characterization of the 0.5 M DQ-DC anolyte using ^{1}H NMR spectroscopy, recorded in D_2O.

We further conducted post-cycling quantitative ^{1}H NMR measurements on DQ-DC collected before and after designed cycling time, using ethylene carbonate as an internal standard (**Figure S23**). Ethylene carbonate was added in the same fixed amount to both samples, allowing direct comparison of resonance integrals across cycles.

We focused on well-resolved proton resonances that are sensitive to the molecular structure of DQ-DC and do not overlap with solvent or electrolyte signals. Specifically, we analyzed the aromatic proton signal in the 8.8-9.0 ppm region and another proton signal in the 8.4-8.6 ppm region. In the pre-cycling sample, the integral of the 8.7-9.0 ppm signal was 3.79, while after cycling it was 3.50. Similarly, the 8.4-8.6 ppm resonance had an integral of 7.99 before cycling and 7.37 after cycling.

From these integral changes, we calculated the apparent daily decay rates for each monitored signal. The proton resonance in the 8.8-9.0 ppm region corresponds to a fade rate of approximately 0.03263% per day, and the proton resonance in the 8.4-8.6 ppm region corresponds to a fade rate of approximately 0.03309% per day. Averaging these values yields an overall molecular degradation rate of 0.03286% per day under the extended cycling conditions studied.

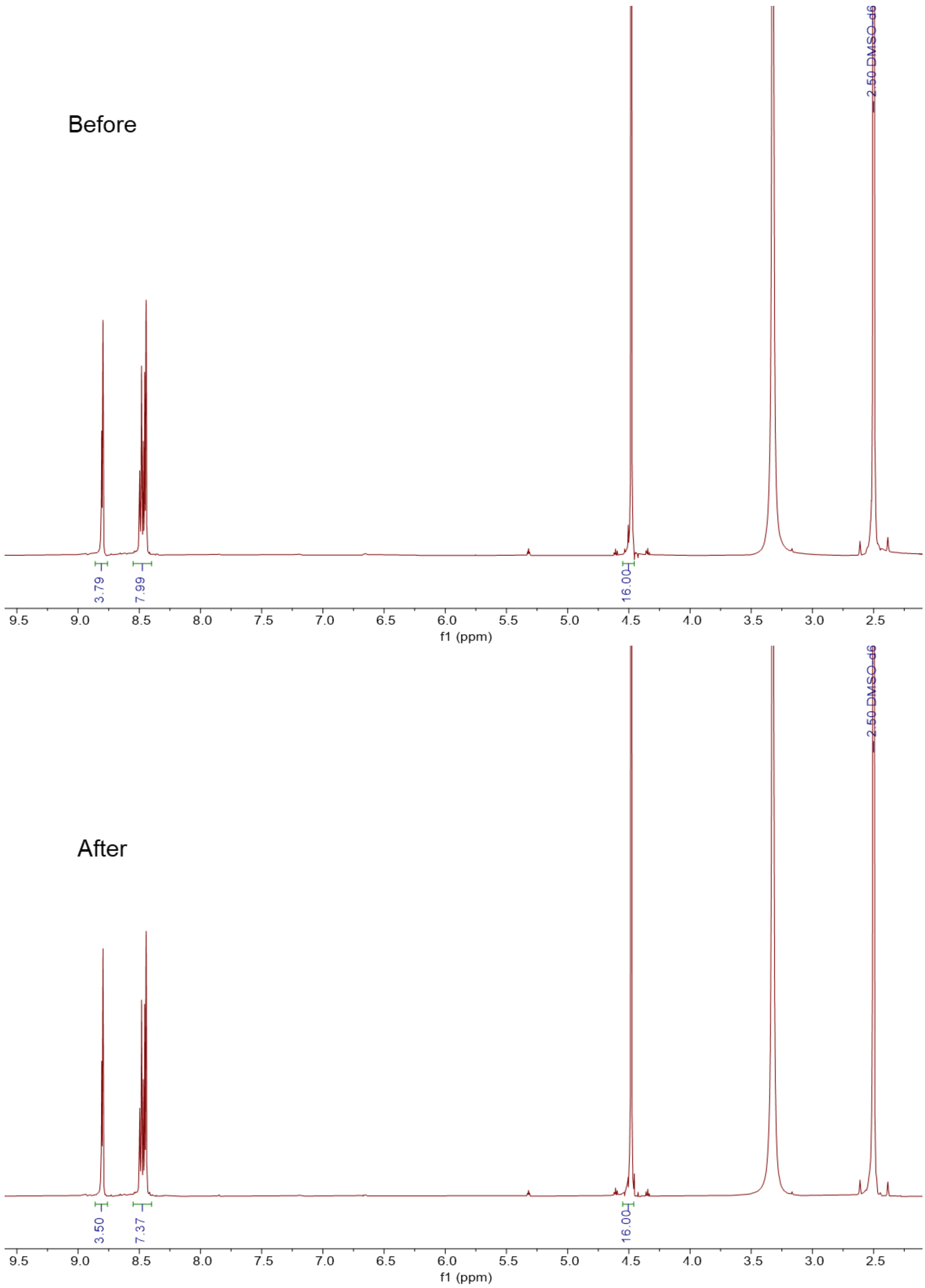

Figure S24. Quantitative NMR analysis of DQ-DC before (top) and after (down) in designed cycling time, with ethylene carbonate added in the same amount, recorded in DMSO-d_6.

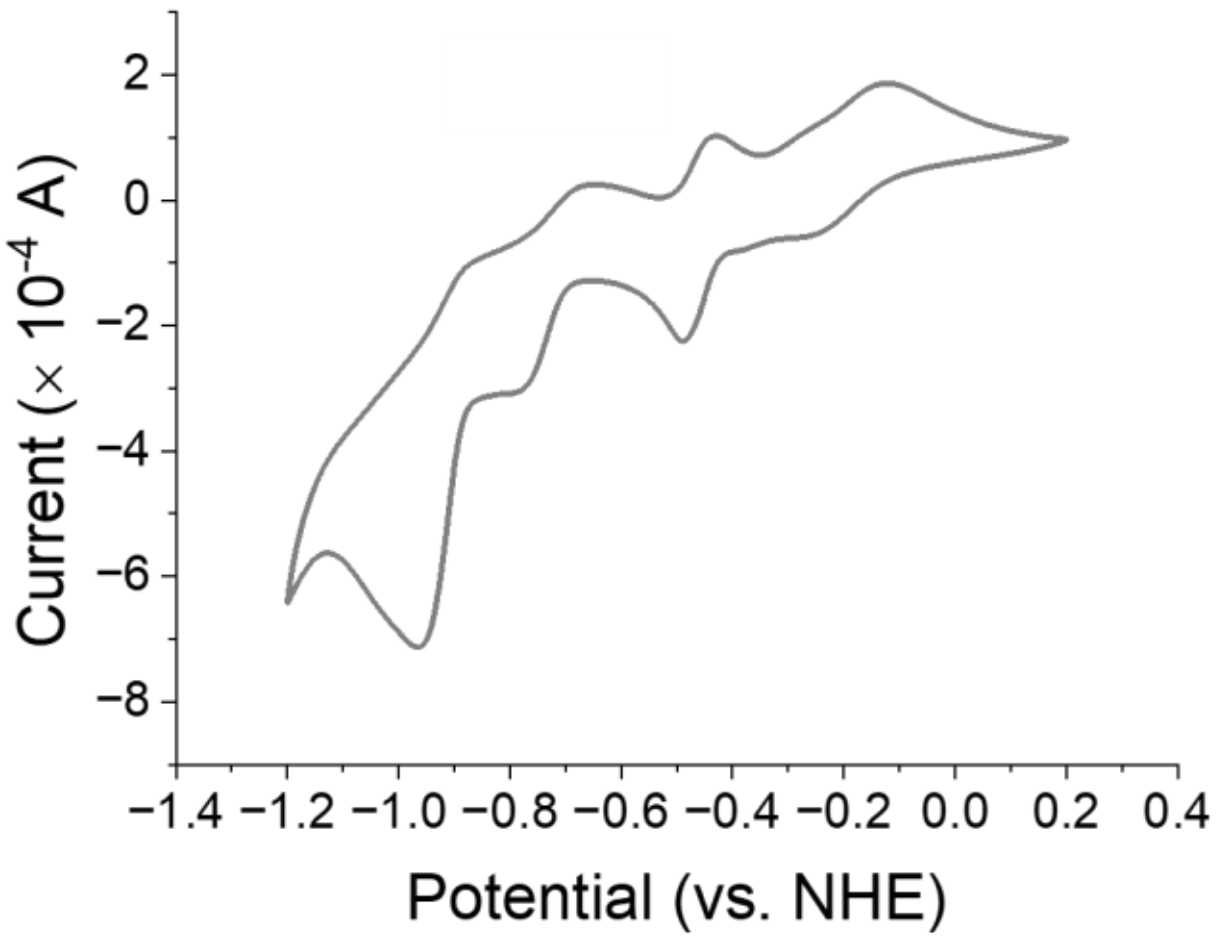

Figure S25. The post-cycle CV profile of DQ-DC anolyte.

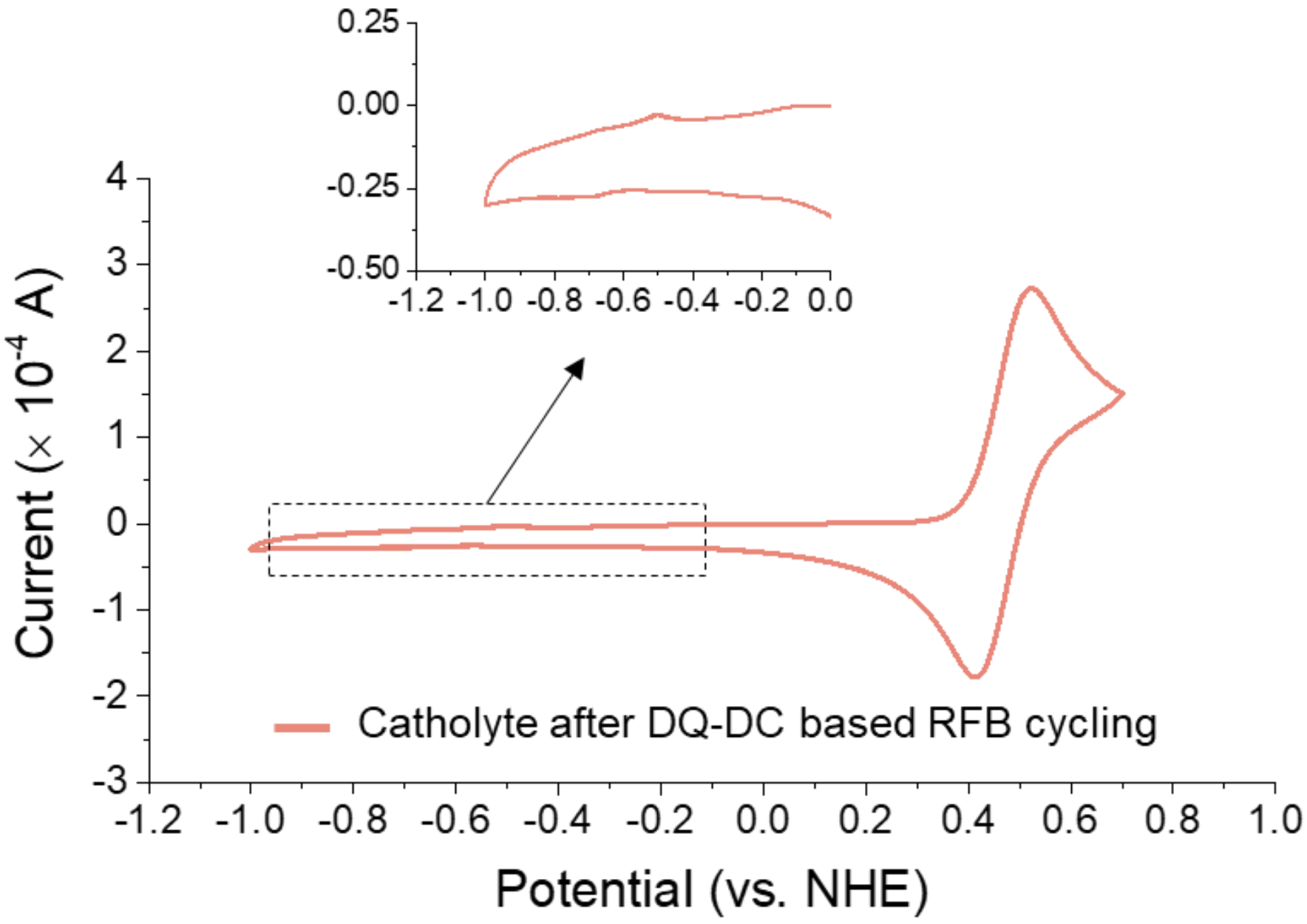

Figure S26. Post-cycling permeability analysis of $K_4Fe(CN)_6$ catholyte in the 0.5 M DQ-DC based RFB using CV method. Inset is the enlarged view of the CV curve.

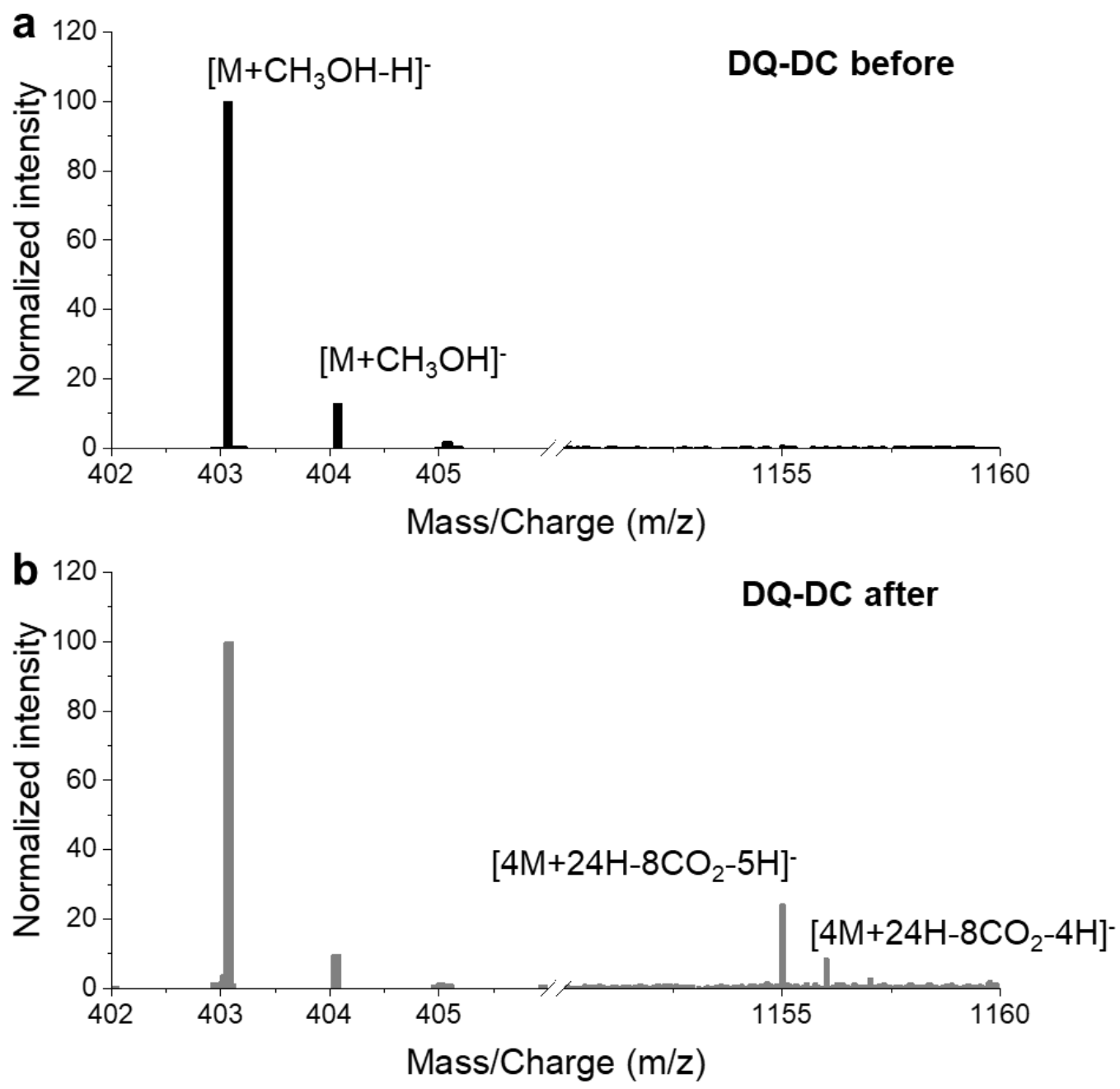

Figure S27. Mass spectrometry analysis of the DQ-DC anolyte before and after battery cycling.

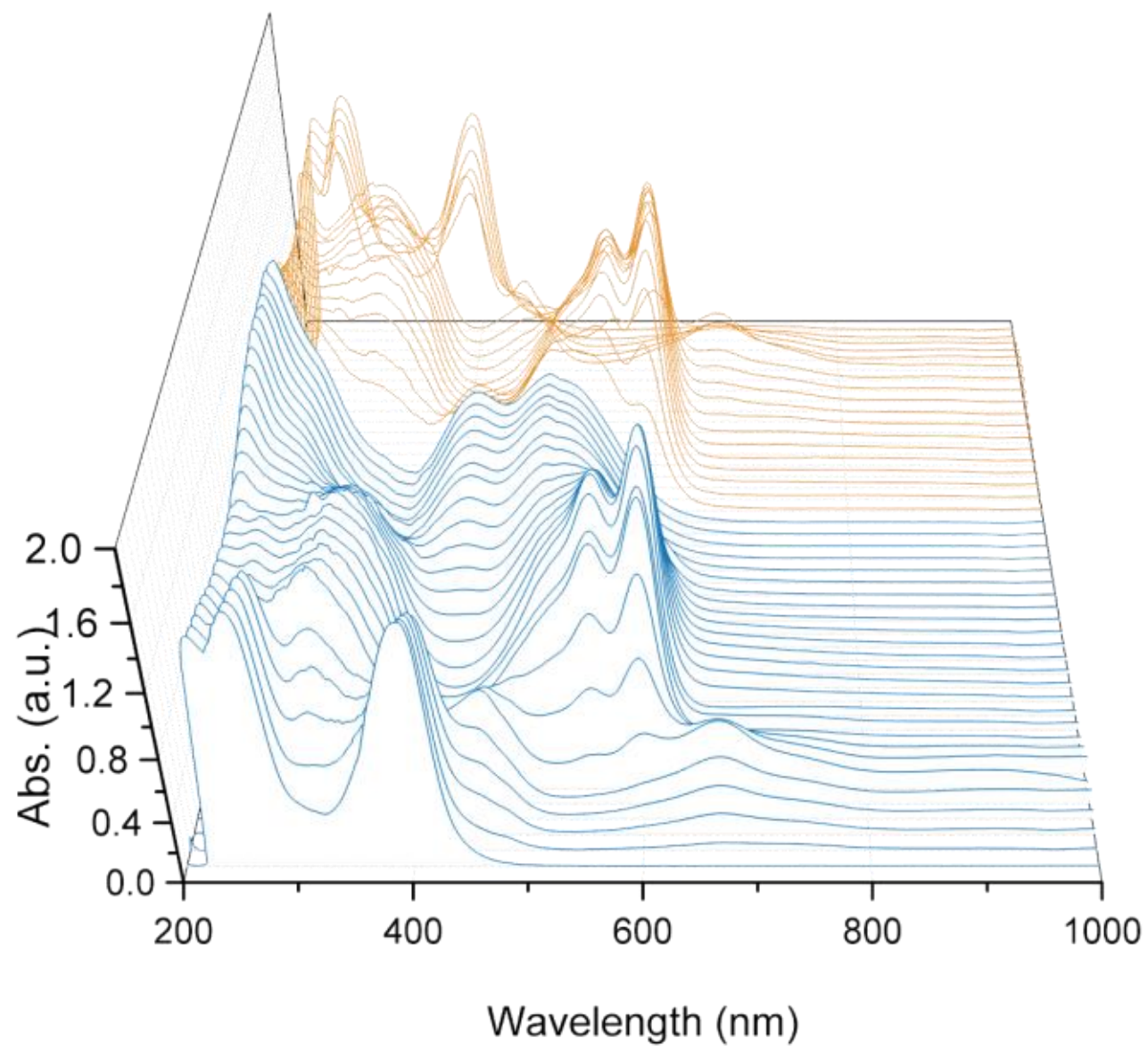

Figure S28. In-situ UV-Vis spectra of the DQ-DC anolyte recorded during stepwise charging from 0% to 100% SOC.

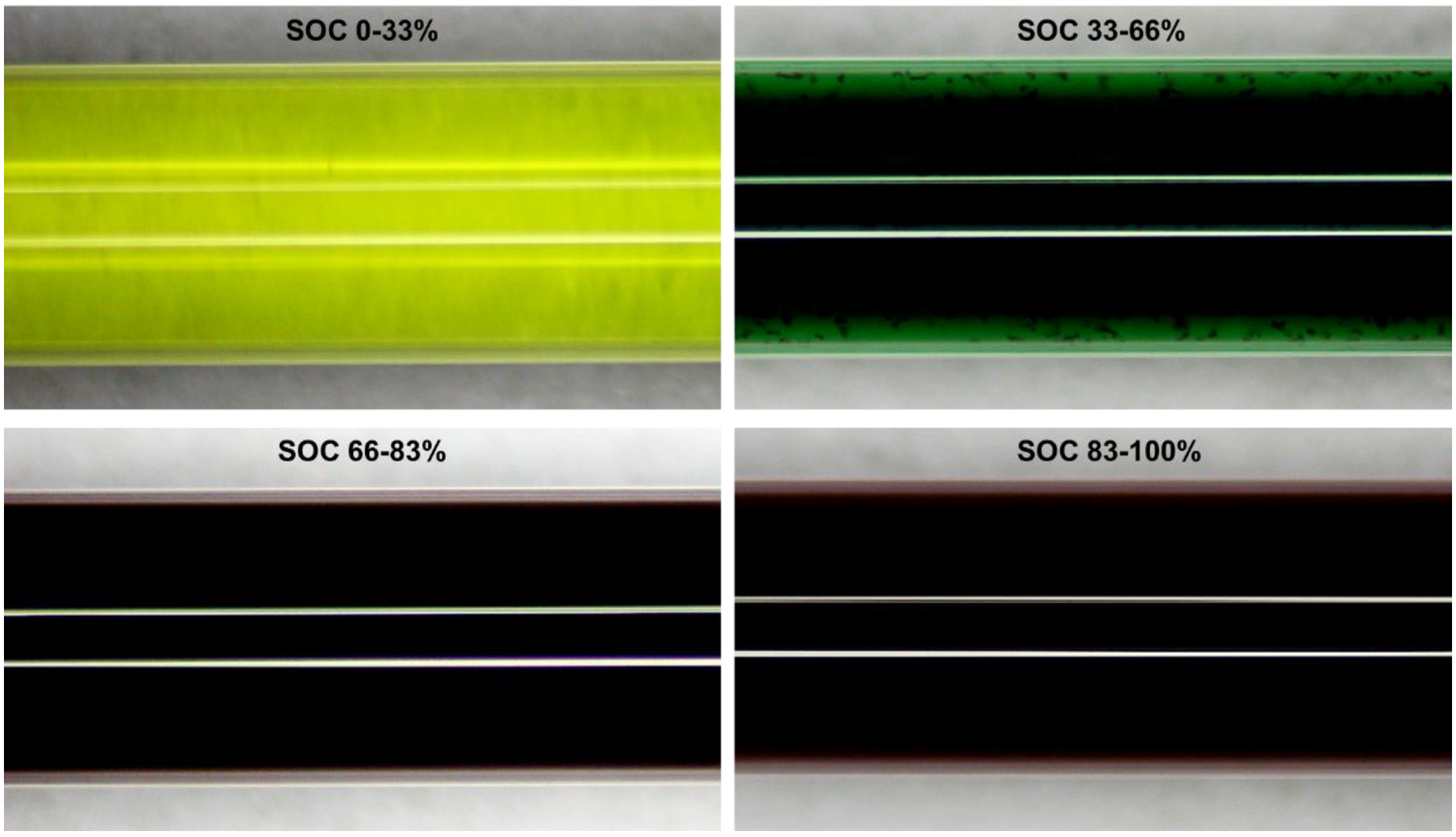

Figure S29. The photographs of DQ-DC in 1.0 M KOH at varying SOCs.

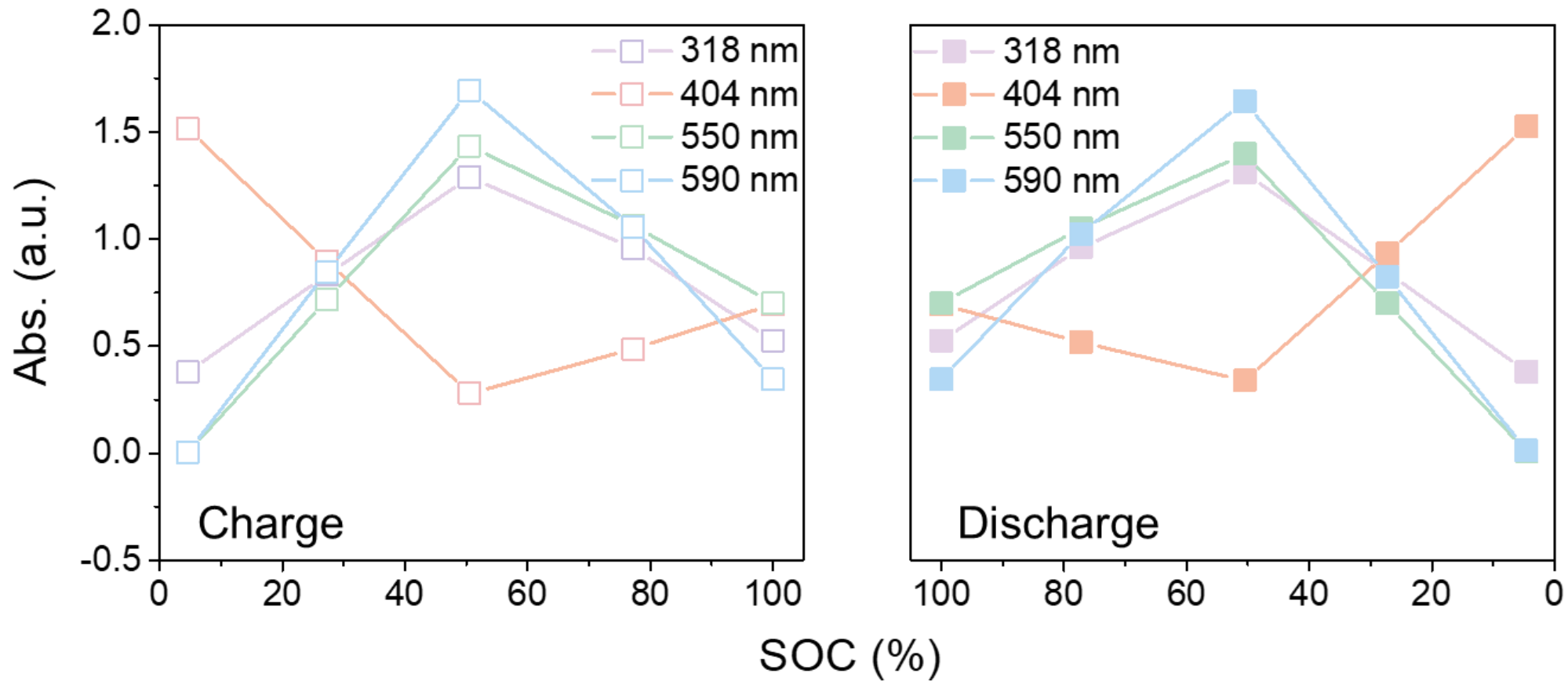

Figure S30. Linearly fitted plot of the absorbance at 318, 404, 550 and 590 nm as a function of the SOC. Below SOC 50%, the absorbance intensities at 318, 550, and 590 nm increase linearly, while the intensity at 404 nm decreases, corresponding to the deepening green color and increasing concentration of DQ-DC-2H. Above 50% SOC, the absorbance at 660 nm decreases reversibly and the intensity at 404 nm rises sharply as the solution color transitions from dark green to red, indicating the reduction of DQ-DC-4H to DQ-DC-6H.

Table S3. The comparison of electrolytes and battery performances of the RFBs.

Anolyte abbreviations	Catholyte abbreviations	Redox potential (V vs. Ag/AgCl)		Cycles (n)	Days (n)	Electron Concentration (M)	Anolyte capacity fade rate (%/cycle)	Anolyte capacity fade rate (%/day)	Refs.
		Anolyte	Catholyte						
DQ-DC	$K_4Fe(CN)_6$	-0.213<br>-0.432 vs.<br>-0.718 NHE<br>-0.899	0.3	3500<br>700	198.5<br>108.6	0.60<br>3.0	0.0023<br>0.0063	0.039<br>0.042	This work
HATNTA		-0.69<br>-0.87<br>-0.94		2000<br>80	46<br>10	0.3<br>1.5	0.0011<br>0.021	0.026<br>0.168	(43)
DHPS		-1.3		1500<br>500	21<br>14.3	0.2<br>2.8	0.0079<br>0.0195	0.93<br>0.68	(38)
FMN-Na		-0.7		200<br>100	6.2<br>~2	0.2<br>0.48	Unavailable<br>0.01	Unavailable<br>0.5	(44)
ACA		-0.9		400	4.5	1	0.02	1.80	(45)
BHPC		-0.9		1000<br>1305	4.23<br>23	0.2<br>1	0.00016<br>0.014	0.04<br>0.08	(46)
1,6-DBAP		-0.76		330<br>90	9<br>7	0.2<br>1	0.00007<br>0.000002	0.0024<br>0.0015	(47)
1,8-PFP		-0.78		~130	40	2	nearly 0	nearly 0	(48)
BTMEP-Vi	TMEPO-OH	-0.54<br>-0.89	0.6	300	0.81	0.2	0.04	15.12	(49)
DQ^{2+}		-0.63		100	0.26	0.2	0.05	19.23	(50)

					(calculated)				
Basic Red 5	$Ce(CH_3SO_3)_3$	0	1.4	200	0.375 (calculated)	0.1	0.0005	0.375 (calculated)	[51]
Vanadium	MB	-0.5	0.3	160<br>50	8<br>5	2.4<br>3.0	0.025<br>0.074	0.52<br>0.76	[52]
	PTO-PTS	-0.5	0.30<br>0.56<br>0.60 vs. SHE	5200	60	4.0	nearly 0	nearly 0	(18)
	BB3	-0.5	0.3	1500	9	0.1	0.009	1.44	[53]

References

1. Peng, C., *et al.* (2017) Reversible multi-electron redox chemistry of π-conjugated N-containing heteroaromatic molecule-based organic cathodes. *Nature Energy*, **2**, 17074.
2. Wang, J., *et al.* (2018) Hexaazatriphenylene derivatives/GO composites as organic cathodes for lithium ion batteries. *J Mater Chem A*, **6**, 2752-2757.
3. Mohebat, R., *et al.* (2017) Synthesis of functionalized γ-spiroiminolactones through a one-pot three-component reaction of isocyanides, acetylenic esters, and 6H-Indeno[1,2-b]pyrido[3,2-e]pyrazin-6-one. *Polycycl Aromat Comp*, **40**, 214-218.
4. Neron, S., *et al.* (2022) Diphenoquinones Redux. *J Org Chem*, **87**, 7673-7695.
5. Huisman, G.W., *et al.* (2010) Practical chiral alcohol manufacture using ketoreductases. *Curr Opin Chem Biol*, **14**, 122-9.
6. Janoschka, T., *et al.* (2016) An aqueous redox-flow battery with high capacity and power: The TEMPTMA/MV system. *Angew Chem Int Ed*, **55**, 14427-14430.
7. Kwabi, D.G., *et al.* (2018) Alkaline quinone flow battery with long lifetime at pH 12. *Joule*, **2**, 1894-1906.
8. Becke, A.D. (1993) Density-functional thermochemistry. III. The role of exact exchange. *J Chem Phys*, **98**, 5648-5652.
9. Krishnan, R., *et al.* (1980) Self-consistent molecular orbital methods. XX. A basis set for correlated wave functions. *J Chem Phys*, **72**, 650-654.
10. Grimme, S., *et al.* (2010) A consistent and accurate ab initio parametrization of density functional dispersion correction (DFT-D) for the 94 elements H-Pu. *J Chem Phys*, **132**, 154104.
11. Marenich, A.V., *et al.* (2009) Universal solvation model based on solute electron density and on a continuum model of the solvent defined by the bulk dielectric constant and atomic surface tensions. *J Phys Chem B*, **113**, 6378-96.
12. Lu, T., *et al.* (2012) Atomic dipole moment corrected hirshfeld population method. *J Theor Comput Chem*, **11**, 163-183.
13. Lu, T., *et al.* (2013) Bond order analysis based on the Laplacian of electron density in fuzzy overlap space. *J Phys Chem A*, **117**, 3100-8.
14. Lu, T., *et al.* (2012) Multiwfn: A multifunctional wavefunction analyzer. *J Comput Chem*, **33**, 580-592.
15. Yamashina, M., *et al.* (2019) An antiaromatic-walled nanospace. *Nature*, **574**, 511-515.
16. Geuenich, D., *et al.* (2005) Anisotropy of the induced current density (ACID), a general method to quantify and visualize electronic delocalization. *Chem Rev* **105**, 3758-3772.
17. Huskinson, B., *et al.* (2014) A metal-free organic-inorganic aqueous flow battery. *Nature*, **505**, 195-198.
18. Ge, G., *et al.* (2025) Four-electron-transferred pyrene-4,5,9,10-tetraone derivatives enabled high-energy-density aqueous organic flow batteries. *J Am Chem Soc*, **147**, 4790-4799.
19. Zhang, P., *et al.* (2025) An amphoteric and hydrogen-bond-rich artificial α-amino acid for highly durable aqueous redox flow batteries. *Nat Commun*, **16**, 4727.
20. Gerhardt, M.R., *et al.* (2016) Anthraquinone derivatives in aqueous flow batteries. *Adv Energy Mater*, **7**, 1601488.
21. Zhang, H., *et al.* (2025) Synergistic ionic modification strategy enhances the stability of naphthalene diimide zwitterions for cost-effective aqueous organic redox flow batteries. *Natl Sci Rev*, DOI: 10.1093/nsr/nwaf123.

22. Wiberg, C., *et al.* (2019) Electrochemical evaluation of a napthalene diimide derivative for potential application in aqueous organic redox flow batteries. *Energy Technol*, **7**, 1900843.
23. Mukhopadhyay, A., *et al.* (2019) Abundant organic dye as an anolyte for aqueous flow battery with multielectron transfer. *ACS Appl Energy Mater*, **2**, 7425-7437.
24. Hong, J., *et al.* (2018) Neutral red and ferroin as reversible and rapid redox materials for redox flow batteries. *ChemSusChem*, **11**, 1866-1872.
25. Tian, Y., *et al.* (2019) Unlocking high-potential non-persistent radical chemistry for semi-aqueous redox batteries. *Chem Commun*, **55**, 2154-2157.
26. Liu, T., *et al.* (2016) A total organic aqueous redox flow battery employing a low cost and sustainable methyl viologen anolyte and 4-HO-TEMPO catholyte. *Adv Energy Mater*, **6**, 1501449.
27. Wang, H., *et al.* (2021) An unsymmetrical two-electron viologens anolyte for salt cavern redox flow battery. *J Power Sources*, **492**, 229659.
28. Liu, B., *et al.* (2020) An aqueous organic redox flow battery employing a trifunctional electroactive compound as anolyte, catholyte and supporting electrolyte. *J Power Sources*, **477**, 228985.
29. Luo, J., *et al.* (2018) A pi-conjugation extended viologen as a two-electron storage anolyte for total organic aqueous redox flow batteries. *Angew Chem Int Ed*, **57**, 231-235.
30. Li, H., *et al.* (2021) Spatial structure regulation: A rod-shaped viologen enables long lifetime in aqueous redox flow batteries. *Angew Chem Int Ed*, **60**, 26971-26977.
31. Janoschka, T., *et al.* (2017) An approach toward replacing vanadium: A single organic molecule for the anode and cathode of an aqueous redox-flow battery. *ChemistryOpen*, **6**, 216-220.
32. Beh, E.S., *et al.* (2017) A neutral pH Aqueous organic–organometallic redox flow battery with extremely high capacity retention. *ACS Energy Lett*, **2**, 639-644.
33. Hu, P., *et al.* (2019) Renewable-lawsone-based sustainable and high-voltage aqueous flow battery. *Energy Storage Mater*, **19**, 62-68.
34. Hu, B., *et al.* (2021) Five-membered ring nitroxide radical: A new class of high-potential, stable catholyte for neutral aqueous organic redox flow batteries. *Adv Funct Mater*, **31**, 2102734.
35. Hu, B., *et al.* (2019) A pH-neutral, metal-free aqueous organic redox flow battery employing an ammonium anthraquinone anolyte. *Angew Chem Int Ed*, **58**, 16629-16636.
36. Feng, R., *et al.* (2021) Reversible ketone hydrogenation and dehydrogenation for aqueous organic redox flow batteries. *Science*, **372**, 836-840.
37. Pang, S., *et al.* (2021) Biomimetic amino acid functionalized phenazine flow batteries with long lifetime at near-neutral pH. *Angew Chem Int Ed*, **60**, 5289-5298.
38. Hollas, A., *et al.* (2018) A biomimetic high-capacity phenazine-based anolyte for aqueous organic redox flow batteries. *Nature Energy*, **3**, 508-514.
39. Wang, C., *et al.* (2020) Molecular design of fused-ring phenazine derivatives for long-cycling alkaline redox flow batteries. *ACS Energy Lett*, **5**, 411-417.
40. Xu, J., *et al.* (2021) Ultrastable aqueous phenazine flow batteries with high capacity operated at elevated temperatures. *Joule*, **5**, 2437-2449.
41. Huang, J., *et al.* (2021) Radical Stabilization of a tripyridinium–triazine molecule enables reversible storage of multiple electrons. *Angew Chem Int Ed*, **60**, 20921-20925.
42. Lin, K., *et al.* (2016) A redox-flow battery with an alloxazine-based organic electrolyte. *Nature Energy*, **1**, 16102.

43. Hu, B., *et al.* (2023) A long-lifetime aqueous organic redox flow battery utilizing multi-redox anolyte. *Energy Storage Mater*, **59**, 102789.
44. Orita, A., *et al.* (2016) A biomimetic redox flow battery based on flavin mononucleotide. *Nat Commun*, **7**, 13230.
45. Lin, K.X., *et al.* (2016) A redox-flow battery with an alloxazine-based organic electrolyte. *Nature Energy*, **1**, 16102.
46. Wang, C.X., *et al.* (2020) Molecular design of fused-ring phenazine derivatives for long-cycling alkaline redox flow batteries. *ACS Energy Lett*, **5**, 411-417.
47. Pang, S., *et al.* (2021) Biomimetic amino acid functionalized phenazine flow batteries with long lifetime at near-neutral pH. *Angew Chem Int Ed*, **60**, 5289-5298.
48. Xu, J.C., *et al.* (2021) Ultrastable aqueous phenazine flow batteries with high capacity operated at elevated temperatures. *Joule*, **5**, 2437-2449.
49. Liu, Y., *et al.* (2025) A highly soluble two-electron storage viologen negolyte for neutral aqueous organic redox flow batteries. *ACS Omega*, **10**, 18641-18649.
50. Lv, Y., *et al.* (2019) Structure reorganization-controlled electron transfer of bipyridine derivatives as organic redox couples. *J Mater Chem A*, **7**, 27016-27022.
51. Lai, Y.Y., *et al.* (2020) Stable low-cost organic dye anolyte for aqueous organic redox flow battery. *ACS Appl Energy Mater*, **3**, 2290-2295.
52. Zhang, C., *et al.* (2019) Phenothiazine-based organic catholyte for high-capacity and long-life aqueous redox flow batteries. *Adv Mater*, **31**, e1901052.
53. Li, H., *et al.* (2020) A stable organic dye catholyte for long-life aqueous flow batteries. *Chem Commun*, **56**, 13824-13827.
